# Large-scale genetic characterization of Parkinson’s disease in the African and African admixed populations

**DOI:** 10.1093/brain/awaf379

**Published:** 2025-10-08

**Authors:** Fulya Akçimen, Kimberly Paquette, Peter Wild Crea, Kathryn Step, Emily Waldo, Mathew J Koretsky, Paula Saffie-Awad, Charles Achoru, Funmilola Taiwo, Simon Ozomma, Gerald Onwuegbuzie, Marzieh Khani, Spencer Grant, Lukman Owolabi, Chiamaka Okereke, Olajumoke Oshinaike, Emmanuel Iwuozo, Suleyman Can Akerman, Paul Suhwan Lee, Shyngle Oyakhire, Nosakhare Osemwegie, Kensuke Daida, Sani Abubakar, Adedunni Olusanya, Mariam Isayan, Christiane Alvarez, Rami Traurig, Adebimpe Ogunmodede, Sarah Samuel, Mary B Makarious, Fadimatu Sa’ad, Rashidat Olanigan, Kristin Levine, Ewere Marie Ogbimi, Dan Vitale, Francis Odiase, Francis Ojini, Olanike Odeniyi, Zih-Hua Fang, Nkechi Obianozie, Deborah A Hall, Ernest Nwazor, Tao Xie, Francesca Nwaokorie, Mahesh Padmanaban, Paul Nwani, Ejaz A Shamim, Alero Nnama, David Standaert, Morenikeji Komolafe, Marissa Dean, Godwin Osaigbovo, Elizabeth Disbrow, Ismaila Ishola, Ashley Rawls, Frank Imarhiagbe, Shivika Chandra, Cyril Erameh, Vanessa Hinson, Naomi Louie, Ahmed Idowu, J Solle, Scott A Norris, Abdullahi Ibrahim, Camilla Kilbane, Gauthaman Sukumar, Lisa M Shulman, Daniel Ezuduemoih, Julia Staisch, Sarah Breaux, Clifton Dalgard, Erin R Foster, Abiodun Bello, Andrew Ameri, Raquel Real, Erica Ikwenu, Huw R Morris, Roosevelt Anyanwu, Erin Furr Stimming, Kimberley Billingsley, Wemimo Alaofin, Pilar Alvarez Jerez, Osigwe Agabi, Dena G Hernandez, Rufus Akinyemi, Sampath Arepalli, Laksh Malik, Raymond Owolabi, Yakub Nyandaiti, Hampton L Leonard, Kolawole Wahab, Oladunni Abiodun, Carlos F Hernandez, Fatima Abdulai, Hirotaka Iwaki, Soraya Bardien, Christine Klein, John Hardy, Henry Houlden, Kamalini Ghosh Galvelis, Mike A Nalls, Nabila Dahodwala, Whitley Aamodt, Emily Hill, Alberto Espay, Stewart Factor, Chantale Branson, Cornelis Blauwendraat, Andrew B Singleton, Oluwadamilola Ojo, Lana M Chahine, Njideka Okubadejo, Sara Bandres-Ciga

**Affiliations:** Laboratory of Neurogenetics, National Institute on Aging, National Institutes of Health, Bethesda, MD 20892, USA; Center for Alzheimer’s and Related Dementias, National Institute on Aging and National Institute of Neurological Disorders and Stroke, National Institutes of Health, Bethesda, MD 20892, USA; Laboratory of Neurogenetics, National Institute on Aging, National Institutes of Health, Bethesda, MD 20892, USA; Center for Alzheimer’s and Related Dementias, National Institute on Aging and National Institute of Neurological Disorders and Stroke, National Institutes of Health, Bethesda, MD 20892, USA; Department of Biomedical Sciences, Division of Molecular Biology and Human Genetics, Faculty of Medicine and Health Sciences, Stellenbosch University, Cape Town 7505, South Africa; South African Medical Research Council Centre for Tuberculosis Research, Stellenbosch University, Cape Town 7505, South Africa; Genomic Medicine, Lerner Research Institute, Cleveland Clinic Foundation, Cleveland, OH 44106, USA; Center for Alzheimer’s and Related Dementias, National Institute on Aging and National Institute of Neurological Disorders and Stroke, National Institutes of Health, Bethesda, MD 20892, USA; DataTecnica LLC, Washington, DC 20037, USA; Programa de Pós-Graduação em Ciências Médicas, Universidade Federal do Rio Grande do Sul, Porto Alegre 90035, Brazil; Clínica Santa María, Santiago 7520378, Chile; Jos University Teaching Hospital, Jos, Plateau State 930105, Nigeria; University College Hospital, Ibadan, Oyo State 200005, Nigeria; Department of Internal Medicine, University of Calabar Teaching Hospital, Calabar, Cross River State 540281, Nigeria; Department of Internal Medicine, University of Abuja, Gwagwalada, Federal Capital Territory 902101, Nigeria; Center for Alzheimer’s and Related Dementias, National Institute on Aging and National Institute of Neurological Disorders and Stroke, National Institutes of Health, Bethesda, MD 20892, USA; Laboratory of Neurogenetics, National Institute on Aging, National Institutes of Health, Bethesda, MD 20892, USA; Center for Alzheimer’s and Related Dementias, National Institute on Aging and National Institute of Neurological Disorders and Stroke, National Institutes of Health, Bethesda, MD 20892, USA; Department of Medicine, Bayero University, Kano, Kano State 700001, Nigeria; Department of Medicine, University of Nigeria Teaching Hospital, Ituku-Ozalla, Enugu State 402109, Nigeria; Department of Medicine, Lagos State University College of Medicine, Ikeja, Lagos State P.M.B. 0001, Nigeria; Department of Internal Medicine, Benue State University, Makurdi, Benue State 970001, Nigeria; Brain Science Institute, Johns Hopkins University School of Medicine, Baltimore, MD 21205, USA; Department of Neurology, Johns Hopkins University School of Medicine, Baltimore, MD 21205, USA; Laboratory of Neurogenetics, National Institute on Aging, National Institutes of Health, Bethesda, MD 20892, USA; Department of Internal Medicine, National Hospital Abuja, Abuja, Federal Capital Territory 900103, Nigeria; Department of Medicine, University of Port Harcourt Teaching Hospital, Port Harcourt, Rivers State 500004, Nigeria; Laboratory of Neurogenetics, National Institute on Aging, National Institutes of Health, Bethesda, MD 20892, USA; Center for Alzheimer’s and Related Dementias, National Institute on Aging and National Institute of Neurological Disorders and Stroke, National Institutes of Health, Bethesda, MD 20892, USA; Department of Neurology, Faculty of Medicine, Juntendo University, Tokyo 113-8421, Japan; Department of Medicine, Ahmadu Bello University, Zaria, Kaduna State 800001, Nigeria; Department of Pharmacology, Therapeutics and Toxicology, College of Medicine University of Lagos, Idi-araba, Lagos State P.M.B 12003, Nigeria; R-Jolad Hospital, Gbagada, Lagos 100254, Nigeria; Department of Neurology and Neurosurgery, National Institute of Health, Yerevan 0051, Armenia; Center for Alzheimer’s and Related Dementias, National Institute on Aging and National Institute of Neurological Disorders and Stroke, National Institutes of Health, Bethesda, MD 20892, USA; Center for Alzheimer’s and Related Dementias, National Institute on Aging and National Institute of Neurological Disorders and Stroke, National Institutes of Health, Bethesda, MD 20892, USA; Neurology Unit, Department of Medicine, Federal Medical Center, Owo, Ondo State 341101, Nigeria; University of Maiduguri Teaching Hospital, Maiduguri, Borno State 600230, Nigeria; Center for Alzheimer’s and Related Dementias, National Institute on Aging and National Institute of Neurological Disorders and Stroke, National Institutes of Health, Bethesda, MD 20892, USA; DataTecnica LLC, Washington, DC 20037, USA; Federal Teaching Hospital, Gombe, Gombe State 760253, Nigeria; Neurology Unit, Department of Medicine, Lagos State University Teaching Hospital, Ikeja, Lagos State 101233, Nigeria; Center for Alzheimer’s and Related Dementias, National Institute on Aging and National Institute of Neurological Disorders and Stroke, National Institutes of Health, Bethesda, MD 20892, USA; DataTecnica LLC, Washington, DC 20037, USA; Department of Medicine, Faculty of Clinical Medicine, Delta State University, Abraka, Delta State 330105, Nigeria; Center for Alzheimer’s and Related Dementias, National Institute on Aging and National Institute of Neurological Disorders and Stroke, National Institutes of Health, Bethesda, MD 20892, USA; DataTecnica LLC, Washington, DC 20037, USA; Department of Medicine, College of Medical Sciences, University of Benin, Benin City, Edo State 300242, Nigeria; Department of Medicine, Ahmadu Bello University, Zaria, Kaduna State 800001, Nigeria; Department of Medicine, Neurology Unit, Lagos University Teaching Hospital, Idi-araba, Lagos State 102215, Nigeria; General Hospital, Lagos Island, Lagos State 102273, Nigeria; German Center for Neurodegenerative Diseases, DZNE, Tübingen 72076, Germany; University of Abuja Teaching Hospital, Gwagwalada, Federal Capital Territory 902101, Nigeria; Department of Neurological Sciences, Rush University Medical Center, Chicago, IL 60612, USA; Department of Medicine, Rivers State University Teaching Hospital, Port Harcourt, Rivers State 500101, Nigeria; University of Chicago Medicine, Department of Neurology, Chicago, IL 60637, USA; Department of Pharmacology, Therapeutics and Toxicology, College of Medicine University of Lagos, Idi-araba, Lagos State P.M.B 12003, Nigeria; University of Chicago Medicine, Department of Neurology, Chicago, IL 60637, USA; Internal Medicine, Faculty of Medicine, Nnamdi Azikiwe University Teaching Hospital, Nnewi, Anambra State 435101, Nigeria; Human Motor Control Section, National Institute of Neurological Disorders and Stroke, Bethesda, MD 20892, USA; Department of Neurology, Mid-Atlantic Permanente Medical Group, Largo, MD 20774, USA; Kaiser Permanente, Mid-Atlantic Permanente Research Institute, Washington, DC 20002, USA; Department of Medicine, University of Port Harcourt Teaching Hospital, Port Harcourt, Rivers State 500004, Nigeria; Department of Neurology, University of Alabama at Birmingham, Birmingham, AL 35294, USA; Department of Medicine, Obafemi Awolowo University, Ile-Ife, Osun State 220282, Nigeria; Department of Neurology, University of Alabama at Birmingham, Birmingham, AL 35294, USA; Jos University Teaching Hospital, Jos, Plateau State 930105, Nigeria; Department of Neurology, LSU Health Shreveport, LSU Health Shreveport Center for Brain Health, Shreveport, LA 71103, USA; Department of Medicine, College of Medical Sciences, University of Benin, Benin City, Edo State 300242, Nigeria; Department of Neurology, University of Florida College of Medicine, Gainesville, Florida 32611, USA; Department of Medicine, Faculty of Clinical Medicine, Delta State University, Abraka, Delta State 330105, Nigeria; Department of Neurology, The University of Texas Health Science Center at Houston, Houston, TX 20036, USA; Department of Medicine, Irrua Specialist Teaching Hospital, Irrua, Edo State 312107, Nigeria; Department of Neurology, Medical University of South Carolina, Charleston, SC 29425, USA; Department of Clinical Research, Michael J Fox Foundation for Parkinson’s Research, New York, NY 10163, USA; Department of Medicine, Obafemi Awolowo University Teaching Hospitals Complex, Ile-Ife, Osun State 220282, Nigeria; Department of Clinical Research, Michael J Fox Foundation for Parkinson’s Research, New York, NY 10163, USA; Department of Neurology, Washington University in St Louis, St Louis, MO 63130, USA; Federal University of Health Sciences Teaching Hospital, Azare, Bauchi State 751102, Nigeria; University Hospital in Cleveland Medical Center, Case Western Reserve University (UH), Cleveland, OH 44106, USA; Department of Anatomy, Physiology and Genetics, School of Medicine, Uniformed Services, Bethesda, MD 20814, USA; Center for Military Precision Health, Uniformed Services University of the Health Sciences, Bethesda, MD 20814, USA; Department of Neurology, University of Maryland, Baltimore, MD 21201, USA; Department of Medicine, Neurology Unit, Lagos University Teaching Hospital, Idi-araba, Lagos State 102215, Nigeria; Ochsner Clinic Foundation, New Orleans, LA 70124, USA; Ochsner Clinic Foundation, New Orleans, LA 70124, USA; Henry M Jackson Foundation for the Advancement of Military Medicine, Uniformed Services University of the Health Sciences, Bethesda, MD 20814, USA; The American Genome Center, Collaborative Health Initiative Research Program, Uniformed Services University of the Health Sciences, Bethesda, MD 20814, USA; Department of Neurology, Washington University in St Louis, St Louis, MO 63130, USA; Division of Neurology, Department of Medicine, University of Ilorin Teaching Hospital, Ilorin, Kwara State 234031, Nigeria; Department of Neurology, Medical University of South Carolina, Charleston, SC 29425, USA; Department of Clinical and Movement Neurosciences, UCL Queen Square Institute of Neurology, London WC1N 3BG, UK; UCL Movement Disorders Centre, University College London, London WC1N 3BG, UK; Department of Medicine, Neurology Unit, Lagos University Teaching Hospital, Idi-araba, Lagos State 102215, Nigeria; Department of Clinical and Movement Neurosciences, UCL Queen Square Institute of Neurology, London WC1N 3BG, UK; National Hospital for Neurology and Neurosurgery, London WC1N 3BG, UK; Department of Neurology, Royal Free Hospital, London NW3 2QG, UK; Department of Pharmacology, Therapeutics and Toxicology, College of Medicine University of Lagos, Idi-araba, Lagos State P.M.B 12003, Nigeria; Department of Neurology, The University of Texas Health Science Center at Houston, Houston, TX 20036, USA; Center for Alzheimer’s and Related Dementias, National Institute on Aging and National Institute of Neurological Disorders and Stroke, National Institutes of Health, Bethesda, MD 20892, USA; Department of Medicine, University of Ilorin, Ilorin, Kwara State 240001, Nigeria; Laboratory of Neurogenetics, National Institute on Aging, National Institutes of Health, Bethesda, MD 20892, USA; Department of Clinical and Movement Neurosciences, UCL Queen Square Institute of Neurology, London WC1N 3BG, UK; Department of Pharmacology, Therapeutics and Toxicology, College of Medicine University of Lagos, Idi-araba, Lagos State P.M.B 12003, Nigeria; Department of Medicine, Neurology Unit, Lagos University Teaching Hospital, Idi-araba, Lagos State 102215, Nigeria; Laboratory of Neurogenetics, National Institute on Aging, National Institutes of Health, Bethesda, MD 20892, USA; Neuroscience and Ageing Research Unit, Institute for Advanced Medical Research and Training, College of Medicine, University of Ibadan, Ibadan, 200212 Oyo State, Nigeria; Laboratory of Neurogenetics, National Institute on Aging, National Institutes of Health, Bethesda, MD 20892, USA; Center for Alzheimer’s and Related Dementias, National Institute on Aging and National Institute of Neurological Disorders and Stroke, National Institutes of Health, Bethesda, MD 20892, USA; Neurology Unit, Department of Medicine, Federal Medical Center, Owo, Ondo State 341101, Nigeria; University of Maiduguri Teaching Hospital, Maiduguri, Borno State 600230, Nigeria; Center for Alzheimer’s and Related Dementias, National Institute on Aging and National Institute of Neurological Disorders and Stroke, National Institutes of Health, Bethesda, MD 20892, USA; DataTecnica LLC, Washington, DC 20037, USA; Department of Medicine, University of Ilorin, Ilorin, Kwara State 240001, Nigeria; General Hospital, Isolo, Lagos State 100263, Nigeria; Universidad del Desarrollo, Centro de Genética y Genómica, Facultad de Medicina Clínica Alemana, Santiago 7610658, Chile; University of Abuja Teaching Hospital, Gwagwalada, Federal Capital Territory 902101, Nigeria; Center for Alzheimer’s and Related Dementias, National Institute on Aging and National Institute of Neurological Disorders and Stroke, National Institutes of Health, Bethesda, MD 20892, USA; DataTecnica LLC, Washington, DC 20037, USA; Department of Biomedical Sciences, Division of Molecular Biology and Human Genetics, Faculty of Medicine and Health Sciences, Stellenbosch University, Cape Town 7505, South Africa; Institute of Neurogenetics and Department of Neurology, University of Lübeck and University Hospital Schleswig-Holstein, Lübeck 23562, Germany; Reta Lila Weston Institute of Neurological Studies, University College London,London WC1N 1PJ, UK; Department of Neuromuscular Diseases, UCL Queen Square Institute of Neurology, London WC1N 3BG, UK; Parkinson’s Foundation, New York, NY 10018, USA; Center for Alzheimer’s and Related Dementias, National Institute on Aging and National Institute of Neurological Disorders and Stroke, National Institutes of Health, Bethesda, MD 20892, USA; DataTecnica LLC, Washington, DC 20037, USA; Department of Neurology, Parkinson's Disease and Movement Disorder Center, University of Pennsylvania, Philadelphia, PA 19104, USA; Department of Neurology, Parkinson's Disease and Movement Disorder Center, University of Pennsylvania, Philadelphia, PA 19104, USA; Department of Neurology, University of Cincinnati, Cincinnati, OH 45221, USA; Department of Neurology, University of Cincinnati, Cincinnati, OH 45221, USA; Department of Neurology, Emory University, Atlanta, GA 30322, USA; Department of Internal Medicine, Morehouse College, Atlanta, GA 30314, USA; Laboratory of Neurogenetics, National Institute on Aging, National Institutes of Health, Bethesda, MD 20892, USA; Center for Alzheimer’s and Related Dementias, National Institute on Aging and National Institute of Neurological Disorders and Stroke, National Institutes of Health, Bethesda, MD 20892, USA; Laboratory of Neurogenetics, National Institute on Aging, National Institutes of Health, Bethesda, MD 20892, USA; Center for Alzheimer’s and Related Dementias, National Institute on Aging and National Institute of Neurological Disorders and Stroke, National Institutes of Health, Bethesda, MD 20892, USA; Department of Pharmacology, Therapeutics and Toxicology, College of Medicine University of Lagos, Idi-araba, Lagos State P.M.B 12003, Nigeria; Department of Medicine, Neurology Unit, Lagos University Teaching Hospital, Idi-araba, Lagos State 102215, Nigeria; Department of Neurology, University of Pittsburgh, Pittsburgh, PA 15213, USA; Department of Pharmacology, Therapeutics and Toxicology, College of Medicine University of Lagos, Idi-araba, Lagos State P.M.B 12003, Nigeria; Department of Medicine, Neurology Unit, Lagos University Teaching Hospital, Idi-araba, Lagos State 102215, Nigeria; Center for Alzheimer’s and Related Dementias, National Institute on Aging and National Institute of Neurological Disorders and Stroke, National Institutes of Health, Bethesda, MD 20892, USA

**Keywords:** genetics, Parkinson’s disease, Black and African American population, African ancestry, disease-causing mutations

## Abstract

Elucidating the genetic contributions to Parkinson’s disease aetiology across diverse ancestries is a critical priority for the development of targeted therapies in a global context. We conducted the largest sequencing characterization of potentially disease-causing, protein-altering and splicing mutations in 710 cases and 11 827 controls from genetically predicted African or African admixed ancestries. We explored copy number variants (CNVs) and runs of homozygosity in prioritized early onset and familial cases. Our study identified rare *GBA1* coding variants to be the most frequent mutations among patients with Parkinson’s disease, with a frequency of 4% in our case cohort.

Of the 18 *GBA1* variants identified, 10 were previously classified as pathogenic or likely pathogenic, four were novel and four were reported as of uncertain clinical significance. The most common known disease-associated *GBA1* variants in the Ashkenazi Jewish and European populations, p.Asn409Ser, p.Leu483Pro, p.Thr408Met and p.Glu365Lys, were not identified among the screened Parkinson’s disease cases of African and African admixed ancestry. Similarly, the European and Asian *LRRK2* disease-causing mutational spectrum, including *LRRK2* p.Gly2019Ser and p.Gly2385Arg genetic risk factors, did not appear to play a major role in Parkinson’s disease aetiology among West African ancestry populations. However, we found three heterozygous novel missense *LRRK2* variants of uncertain significance, with two (p.Glu268Ala and p.Arg1538Cys) displaying higher frequencies in the African ancestry population reference datasets. Structural variant analyses revealed the presence of *PRKN* CNVs with a frequency of 0.7% in African and African admixed cases, with 66% of CNVs detected being compound heterozygous or homozygous in early-onset cases, providing further insights into the genetic underpinnings in early-onset juvenile Parkinson’s disease in these populations. Short tandem repeat analysis also identified *ATXN3* CAG repeat expansions within the pathogenic range (CAG_n_ > 45) in three patients with Parkinson’s disease of African ancestry. Novel genetic variation among screened genes warrants further replication and functional prioritization to unravel their pathogenic potential.

Here, we created the most comprehensive genetic catalogue of both known and novel coding and splicing variants potentially linked to Parkinson’s disease aetiology in an underserved population and further conducted global and local ancestry analyses to further explore population-specific effects. Our study has the potential to guide the development of targeted therapies in the emerging era of precision medicine. By expanding genetics research to involve underrepresented populations, we hope that future Parkinson’s disease treatments are not only effective but also inclusive, addressing the needs of diverse ancestral groups.

## Teaser

Most Parkinson’s disease research has focused on European and Asian populations, leaving gaps in understanding the disease in other groups. To help address this imbalance, Akçimen *et al*. analysed genetic data from more than 700 people with Parkinson’s disease and nearly 12 000 controls of African or African admixed ancestry.

## Introduction

Parkinson’s disease (PD) is a multifaceted neurodegenerative disorder influenced by genetics, environment and other factors. The influence of genetics has been primarily studied in European populations, limiting our understanding of the genetic landscape of PD in the African and African admixed populations.^1^ Bridging this gap is essential for advancing equitable precision medicine interventions and developing universally effective strategies for the prevention and treatment of PD.

Genetic risk factors and their impact on PD in individuals of African and African admixed genetic ancestry remain largely unknown. Around 77% of African genetic studies in PD have been conducted in North Africa (mainly Tunisia) and South Africa.^2^ In contrast, there have been limited PD genetic studies in populations originating from central, eastern and the French-speaking west coast of Africa. Similarly, the genetics of African admixed individuals have been poorly studied in the context of PD genetics, with few published studies.^3-5^ As a result, our understanding of the role of genetics in disease aetiology among African admixed individuals lags behind that of European ancestry individuals. Further research with more diverse representation and standardization is crucial for drawing accurate conclusions on a global scale.

The first genome-wide association study (GWAS) exploring PD genetic risk in African and African admixed populations nominated a novel non-coding PD-associated variant (rs3115534) in *GBA1.*^6^ This variant is present in 33% and 22% of the African and African admixed PD cohorts, respectively,^6^ while it is almost completely absent in predominantly European ancestry cohorts. The rs3115534 intronic variant acts by interfering with the splicing of functional *GBA1* transcripts, resulting in reduced glucocerebrosidase activity. This represents a novel mechanism of *GBA1*-derived PD risk and an attractive candidate for precision-based therapeutics in a remarkably underserved population,^7^ reinforcing the idea that distinct genetic architectures contribute to disease susceptibility and, in turn, could inform optimized treatment options in the future. Other *GBA1* coding variants, like p.Asn409Ser and p.Leu483Pro, are associated with PD risk and commonly observed in European and Ashkenazi Jewish ancestry populations. These same variants are notably rare among individuals of African and African admixed ancestries.^8,9^

To gain a deeper understanding of the genetic architecture of PD in underserved African populations, we leveraged data from the Black African and African American Connections to Parkinson’s Disease Study (BLAAC PD)^10^ as a part of the Global Parkinson’s Genetics Program (GP2),^11^ the Nigerian Parkinson’s Disease Research Network (NPDRN),^12^ the PDGENEration (PDGENE) initiative,^13^ All of Us^14^ and the UK Biobank (UKB).^15^ Using data from 710 cases and 11 827 controls, we conducted the largest sequencing characterization of potentially disease-causing, protein-altering and splicing mutations across 51 genes in African and African admixed ancestry populations. We focused on known PD-associated genes linked to autosomal dominant inheritance (*SNCA*, *LRRK2* and *VPS35*), autosomal recessive inheritance (*PRKN*, *PINK1*, *DJ-1* and *VPS13C*) and increased risk (*LRRK2*, *GBA1*), as well as other PD-associated genes such as *RAB32, PSMF1* and *ITSN1* from literature. Furthermore, we assessed putative pleiotropic effects by examining genes that have been shown to manifest as parkinsonism^16^ (i.e. *ATP13A2*, *FBXO7*, *PLA2G6*, *SYNJ1*, *GCH1*, *TRPM7*, *GCH1*, *KCDT7*, *XPR1* and *TOR1A*) or other neurodegenerative conditions (i.e. *APP*, *MAPT* and *DCTN1*). We leveraged short-read sequencing datasets (whole-genome, whole-exome and targeted-exome sequencing) to comprehensively unravel the genetic architecture of rare variants contributing to PD in these populations and further explored potential population-specific effects through global and local ancestry analyses. In addition, we examined short tandem repeats (STRs) previously reported to be associated with PD risk, including *ATXN2*, *ATXN3* and *TBP.*^17-19^ We further investigated potential recessive variants by exploring runs of homozygosity (ROHs) enriched in the patient population. We assessed potential copy number variation (CNVs) by investigating B allele frequency (BAF) and log2 ratio (L2R) from genotyping data along with multiplex ligand-dependent probe amplification (MLPA), focusing on cases with positive family history and early age at onset.

## Materials and methods

### Participants and study design

**Figure 1:**
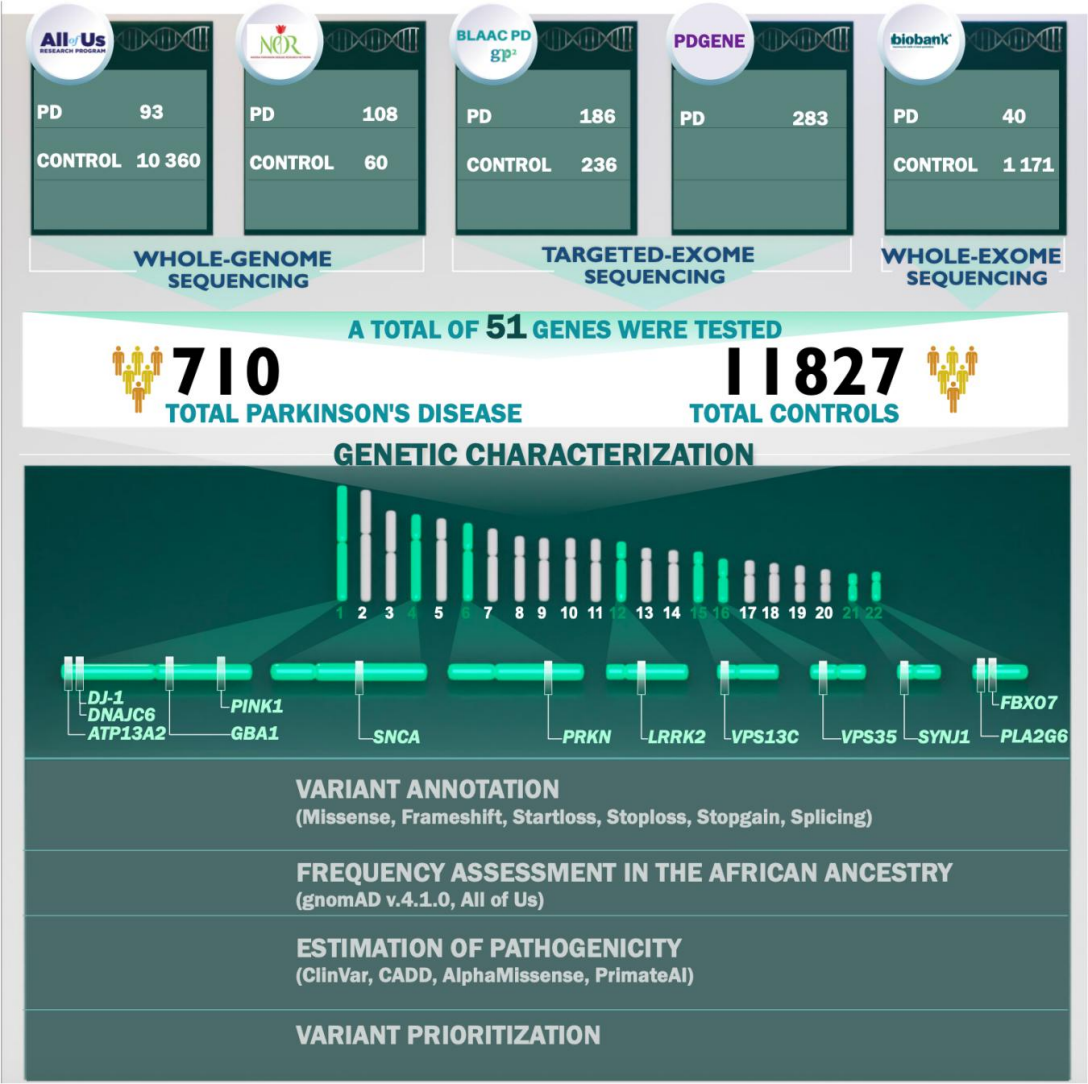
**Workflow.** Our workflow begins with creating cohorts within the datasets. We used short-read sequencing data to characterize Parkinson’s disease, parkinsonism and related neurodegenerative disease genes. We prioritized missense, frameshift, start loss, stop loss, stop gain and splicing variants with a minor allele frequency < 0.01 and Combined Annotation Dependent Depletion (CADD) score > 12.37.

We obtained genetic data from All of Us, GP2-BLAAC PD, the NPDRN, PDGENE and UKB (Supplementary Table 1). In total, the data comprised 710 cases and 11 827 controls of African or African admixed ancestry or Black or African American self-reported race. See Supplementary Table 1 for demographic characteristics of cohorts under study and Fig. 1 for a summary of our workflow, explained in further detail later.

BLAAC PD is a multicentre study in North America recruiting individuals who self-identify as Black and/or African American. In the GP2-BLAAC PD dataset, cases were defined as individuals older than 18 years in the USA with a clinician-confirmed PD diagnosis (*n* = 186), whereas controls were neurologically healthy individuals with no personal or family history of any neurodegenerative disease (*n* = 236). Institutional Review Board (IRB) approval has been provided by each of the individual sites’ IRBs and continuing review by the IRB is conducted annually.^20^ Assessments of cognitive status and clinical features were conducted and reported by respective clinicians. Cognitive status was determined by the cognition component of the Clinical Impression of Severity Index for PD (CISI-PD) with scores ranging from 0 to 6: 0 (normal), 1 (slowness and/or minimal cognitive problems), 2 (mild cognitive problems; no limitations), 3 (mild to moderate cognitive problems; does not need help for basic activities of daily living), 4 (moderate cognitive problems; help is required for some basic activities of daily living), 5 (severe cognitive problems; help is required for most or all basic activities of daily living) and 6 (severely disabled; helpless; complete assistance needed).^21^ Clinical symptoms such as bradykinesia, rest tremor, rigidity and use/response to levodopa was assessed through the International Parkinson and Movement Disorder Society (MDS) Clinical Diagnostic Criteria for idiopathic PD (MDS-PD criteria).^22^

For the NPDRN cohort (cases = 108; controls = 60), PD diagnosis was based on the UK PD Society Brain Bank criteria (excluding the requirement of not more than one affected relative).^12,23^ Study assessments include the Movement Disorders Society Unified Parkinson’s Disease Rating Scale (MDS-UPDRS), a one question, self-reported olfactory assessment and a neurological examination, performed by a study neurologist. Controls were excluded if any neurological signs were present.^12^ All participants provided written informed consent.

In addition, we mined existing data from PDGENE, a multicentre, observational study (NCT04057794, NCT04994015) offering Clinical Laboratory Improvement Amendments (CLIA)-certified genetic testing and genetic counselling to PD patients in North America.^13^ We included targeted exome sequencing data from 138 PD cases with genetically predicted African or African admixed ancestry and 145 self-reported Black or African American PD cases from the GP2 release 8. The study was approved by IRBs, and the Scientific Review and Executive Committees of the Parkinson Study Group. All participants signed informed consent forms.

In the All of Us dataset, we created a case-control cohort comprising 93 PD cases and 10 360 controls of African or African admixed ancestry. Controls were individuals aged 60 years or older with no family history of neurological disease and no present neurological condition in their electronic health records. All participants provided written informed consent. The All of Us IRB reviews the protocol, informed consent and other materials for participants.

PD cases of genetically predicted African ancestry in the UKB (*n* = 40) were defined by the UKB field ID 42032, using diagnoses according to the UKB’s algorithmically defined outcomes v2.0 (https://biobank.ndph.ox.ac.uk/ukb/refer.cgi?id=460). The control cohort (*n* = 1171) includes individuals aged 60 years or older without personal or family history of any neurological disorders.

Sequencing, genotyping procedures, and quality assessment of 710 cases and 11 827 controls are detailed in the Supplementary material, methods.

### Genetic characterization of potentially rare disease-causing variants

We performed functional annotation of identified variants using Annotate Variation (ANNOVAR),^24^ incorporating relevant datasets and prediction tools, and gnomAD v4.1. To distinguish *GBA1* variation from *GBAP1* pseudogene mutations, we implemented the Gauchian targeted caller^25^ on whole-genome sequencing data to detect known *GBAP1* variation within the exons 9–11 homology region. The clinical significance of candidate variants was cross-checked on the ClinVar. Genes implicated in parkinsonism and related neurodegenerative diseases were annotated using the OMIM genemap2 file (downloaded from omim.org in September 2024). We focused on missense, loss-of-function variants including stopgain, stop loss, frameshift, start loss, splice site changes, and short insertions and deletions, with a minor allele frequency below 0.01 and a minimum Combined Annotation Dependent Depletion (CADD) PHRED (a Phred-scaled measure of predicted deleteriousness)-scaled score of 12.37. This threshold is predicted to be pathogenic by Amendola *et al*.^26^ and falls within the top 5%–6% most deleterious variants in the genome, according to Kircher *et al*.^27^ We assigned a confidence level for PD genes using the criteria described by Blauwendraat *et al*.^28^

## Results

### Baseline characteristics

An overview of our study is illustrated in Fig. 1. Demographic and clinical characteristics of five datasets including 710 cases and 11 827 neurological healthy controls are summarized in Supplementary Table 1. All participants were of African and African admixed ancestry based on genetic prediction, except for the PDGENE dataset, where 138 participants genotyped on the NeuroBooster array were confirmed as African or African admixed, and 145 participants were included based on self-reported Black or African American race (Supplementary Fig. 1).

### Genetic findings

We identified 443 rare variants, including 105 in eight PD genes and 338 in genes associated with parkinsonism or other neurodegenerative diseases (Supplementary Table 2). A total of 19 variants were previously reported as pathogenic or likely pathogenic in ClinVar, while 182 variants were novel. A total of 242 variants were reported as benign, likely benign or of uncertain clinical significance. The ClinVar dataset is largely based on mutation reports from European populations and so is likely to have incomplete information on pathogenicity in African individuals. Variants were prioritized based on clinical relevance, zygosity and the presence of early disease onset in cases involving recessive PD genes. This process identified 84 variants across 14% of the cases (*n* = 97) that had no prior classification as benign (Supplementary Table 3).

**Figure 2:**
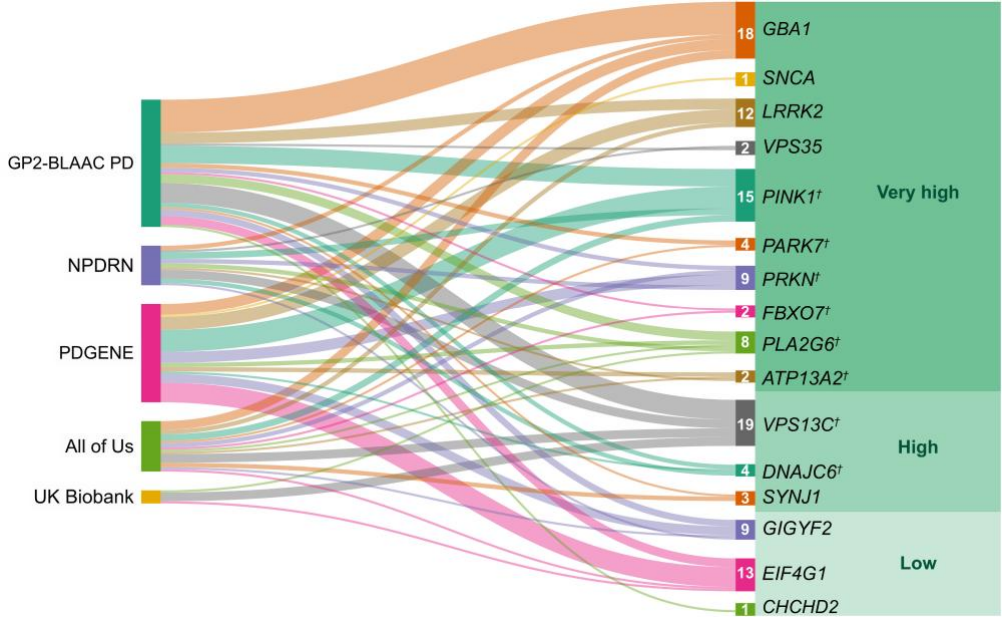
**Distribution of variants in Parkinson’s disease and parkinsonism-related genes.** Parkinson’s disease and parkinsonism-related genes included for analysis. ^†^Autosomal recessive inheritance. ‘Very high’ indicates genes with very high confidence being related to disease, ‘High’ indicates high confidence and ‘Low’ indicates low confidence based on replication in the literature.^28^ Five cohorts were included: Black African and African American Connections to Parkinson’s Disease Study (BLAAC PD), as a part of the Global Parkinson’s Genetics Program (GP2), Nigerian Parkinson’s Disease Research Network (NPDRN), the PD GENEration (PDGENE)ration initiative, the All of US Program and the UK Biobank (UKB). The width of the connecting line between the cohort and each gene is correlated to the number of variants found.

The average age at onset (AAO; mean ± standard deviation) for cases in our study ranged from 56.34 ± 11.90 years (NPDRN) to 66.79 ± 9.55 years (UKB). Among carriers of prioritized variants, the AAO was notably younger at 52.41 ± 13.65 years. Furthermore, carriers with a family history of PD had an earlier AAO of 44.43 ± 13.77 years, compared with 53.64 ± 13.73 years for those without a family history. The distribution of variants in PD genes across the five datasets, excluding those previously reported as benign or likely benign, is presented in Fig. 2.

**Figure 3:**
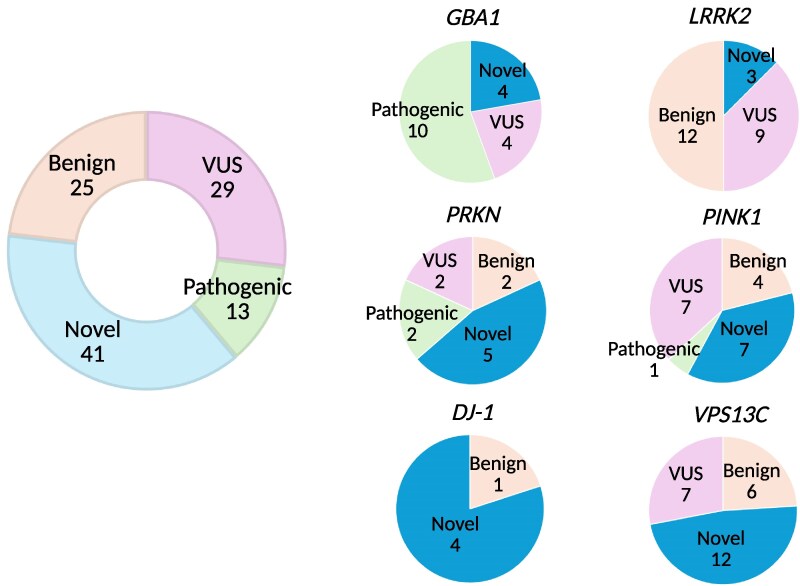
**Prioritized rare protein-altering or splicing variants in known autosomal dominant, recessive and genetic risk factors for Parkinson’s disease**. Autosomal recessive genes for Parkinson’s disease include: *PRKN, PINK1, DJ-1* and *VPS13C*. Genetic risk factors for Parkinson’s disease include: *GBA1* and *LRRK2*. Autosomal dominant genes for Parkinson’s disease include: *SNCA* and *VPS35*. For the latter group, these genes were not individually plotted since only one variant in each autosomal dominant gene was detected. Rare variants were included if minor allele frequency was lower than 0.01 and Combined Annotation Dependent Depletion (CADD) score > 12.37. ‘Novel’ indicates variants not previously reported in the literature. Likely benign and benign variants, as well as likely pathogenic and pathogenic variants according to pathogenicity predictors, are presented together for each gene. VUS = variant of uncertain significance. (Novel variants in other Parkinson’s disease genes include two in *DNAJC6*, one in *SNCA*, two in *VPS35* and one in *SYNJ1.*)

Ten variants in *GBA1*, one in *PINK1*, two in *PRKN*, one in *FBXO7* and one in *PLA2G6* were previously described as pathogenic or likely pathogenic (Fig. 3 and Supplementary Table 3). A total of 41 novel variants were identified in PD genes, including four in *GBA1*, seven in *PINK1*, two in *DNAJC6*, four in *DJ-1*, one in *SNCA*, five in *PRKN*, three in *LRRK2*, 12 in *VPS13C*, two in *VPS35* and one in *SYNJ1* (Fig. 3, Table 2 and Supplementary Table 3). Notably, the *GBA1* p.His413Arg, *SNCA* p.Met116Ile and *PRKN* p.Gly139Valfs*38 have previously been reported only in individuals of African ancestry in the gnomAD and All of Us datasets (Table 1).

**Table 1:** Known pathogenic or likely pathogenic variants identified in Parkinson’s disease genes

Variant	Protein change	Gene	dbSNP	gnomAD AF (AFR)	gnomAD AF (ALL)	Genotype (cases)	Dataset	Ancestry	Sex	Age range	Family history
1:155235002:C:T	p.Arg535His	*GBA1*	rs75822236	5.0 × 10^−5^	1.1 × 10^−4^	1 het	BLAACPD	AFR	Female	70s	No
1:155235196:G:A	p.Arg502Cys	*GBA1*	rs80356771	8.1 × 10^−5^	2.3 × 10^4^	1 het	BLAACPD	AAC	Female	Late 50s	No
1:155235217:C:G	p.Ala495Pro	*GBA1*	rs368060	9.0 × 10^−4^	6.0 × 10^−4^	1 het	All of Us	AFR	Female	Late 50s	NA
1:155235727:C:G	p.Asp448His	*GBA1*	rs1064651	2.5 × 10^−4^	1.1 × 10^−4^	1 het	BLAACPD	AAC	Female	Early 50s	NA
1:155235823:C:T	p.Gly416Ser	*GBA1*	rs121908311	0	2.2 × 10^−5^	1 het	All of Us	AFR	Female	Early 50s	NA
1:155238215:T:C	p.Asn227Ser	*GBA1*	rs364897	2.0 × 10^−4^	6.2 × 10^−5^	1 het	BLAACPD	AFR	Male	Early 50s	NA
1:155238228:A:G	p.Trp223Arg	*GBA1*	rs61748906	1.3 × 10^−5^	1.2 × 10^−5^	1 het	NPDRN	AFR	Male	Early 60s	No
1:155238291:G:A	p.Arg202Ter	*GBA1*	rs1009850780	1.4 × 10^−5^	1.1 × 10^−5^	1 het	PDGENE	AFR	Female	Late 50s	No
1:155238596:C:A	p.Arg170Leu	*GBA1*	rs80356763	0	6.2 × 10^−7^	2 hets	BLAACPD	AFR, AFR	Female	Two siblings, early 30 s and early 40s	Yes, siblings
1:155239968:GGTA:G	p.Thr75del	*GBA1*	rs761621516	5.5 × 10^−4^	3.2 × 10^−5^	3 hets	BLAACPD (1) PDGENE (2)	AFR; AFR; NA	Female in BLAACPD, two males in PDGENE	50s in BLAACPD; 50 and 70s in PDGENE	NA
1:20645615:G:A	p.Ala339Thr	*PINK1*	rs55831733	3.7 × 10^−4^	1.3 × 10^−3^	1 het	PDGENE	NA	Male	50s	No
6:162262560:TCAGTGTGCAGAATGACAGCCAGCCCCACAGAGTCTCCTGG:T	p.Pro113Thrfs*51	*PRKN*	rs771529549	4.0 × 10^−5^	2.6 × 10^−4^	1 het	PDGENE	AFR	Female	20s	No
6:162443325:AT:A	p.Asn52Metfs*29	*PRKN*	rs754809877	6.7 × 10^−5^	2.8 × 10^−4^	2 hets	PDGENE; All of Us	NA; AFR	Female; male	20s; 60s	No
22:32491175:C:T	p.Arg321Ter	*FBXO7*	rs369105683	1.3 × 10^−5^	3.3 × 10^−5^	1 het	PDGENE	AAC	Female	Late 40s	No
22:38132952:G:A	p.Thr319Met	*PLA2G6*	rs149653983	6.2 × 10^−4^	2.9 × 10^−4^	1 het	PDGENE	AFR	Female	Early 50s	No

dbSNP = The Single Nucleotide Polymorphism database; AF = allele frequency in gnomad v4.1; AFR = African/African American; BLAACPD = the Black African and African American Connections to Parkinson's Disease Study; PDGENE = PD GENEration; NPDRN = Nigerian Parkinson’s Disease Research Network; het = heterozygous; AAC = African admixed ancestry; NA = not present; Variant position, referred to hg38. We have received an exception to the Data and Statistics Dissemination Policy from the All of Us Resource Access Board.

**Table 2:** Novel variants identified in Parkinson’s disease genes

Variant	Protein change	Gene	dbSNP	gnomAD AF (AFR)	gnomAD AF (ALL)	Genotype (cases)	Dataset	Ancestry	Inferred ancestry window for the variant	Sex	Age range	Family history
1:155235831:T:C	p.His413Arg	*GBA1*	rs911331923	4.0 × 10^−5^	1.9 × 10^−6^	1 het	BLAACPD	CAH	AFR	Male	40–49	NA
1:155236249:A:C	p.Ile407Ser	*GBA1*	rs1057519358	NA	NA	1 het	BLAACPD	AAC	Unconfirmed (either AFR or EUR)	Male	50–59	No
1:155236379:C:G	p.Gly364Arg	*GBA1*	NA	NA	NA	1 het	BLAACPD	AFR	AFR	Male	50–59	No
1:155237441:GC:G	p.Ala300Profs*4	*GBA1*	NA	NA	NA	1 het	BLAACPD	AFR	AFR	Female	40–49	Yes, 2nd degree relative
4:89729236:C:A	p.Met116Ile	*SNCA*	rs1378041201	NA	NA	1 het	PDGENE	AFR	AFR	Male	50–59	No
6:162201248:AC:A	p.Gly139Valfs*38	*PRKN*	rs751790069	4.0 × 10^−5^	1.9 × 10^−6^	1 het	PDGENE	AFR	NA	Female	20–29	No
12:40243646:A:C	p.Glu268Ala	*LRRK2*	rs373254349	1.6 × 10^−4^	8.1 × 10^−6^	1 het	PDGENE	NA	NA	Female	60–69	No
12:40309228:A:T	p.Ile1438Lys	*LRRK2*	NA	NA	NA	1 het	PDGENE	NA	NA	Female	60–69	No
12:40314047:C:T	p.Arg1538Cys	*LRRK2*	rs150620977	5.6 × 10^−4^	3.1 × 10^−5^	1 het	All of Us	AFR	NA	Female	40–49	No
16:46662991:T:C	p.Met607Val	*VPS35*	rs1555523076	0	6.2 × 10^−7^	1 het	NPDRN	AFR	AFR	Male	40–49	Yes, 2nd degree relative
16:46679050:C:G	p.Asp205His	*VPS35*	NA	NA	NA	1 het	BLAACPD	AAC	AFR	Male	40–49	No

AAC = African admixed ancestry; AF = allele frequency in gnomAD v4.1; AFR = African/African American; BLAACPD = the Black African and African American Connections to Parkinson's Disease Study; CAH = complex admixed ancestry; dbSNP = The Single Nucleotide Polymorphism database; het = heterozygous; NA = not present; NPDRN = Nigerian Parkinson’s Disease Research Network; PDGENE = PD GENEration; Variant position: referred to hg38. We have received an exception to the Data and Statistics Dissemination Policy from the All of Us Resource Access Board.

**Figure 4:**
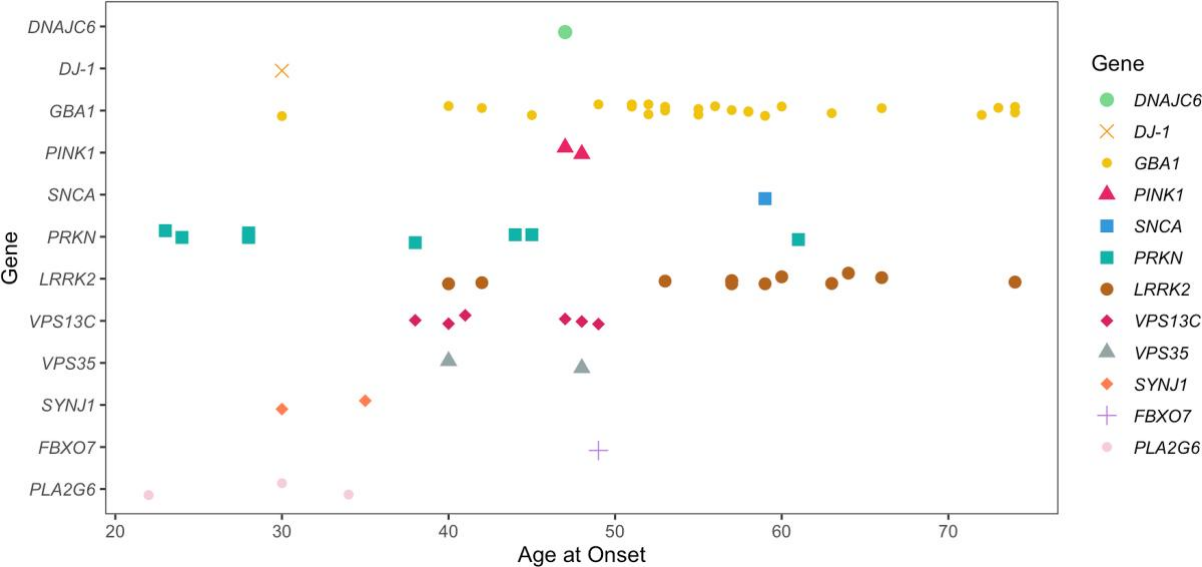
**Distribution of age at onset among Parkinson’s disease prioritized variant carriers**.

**Figure 5:**
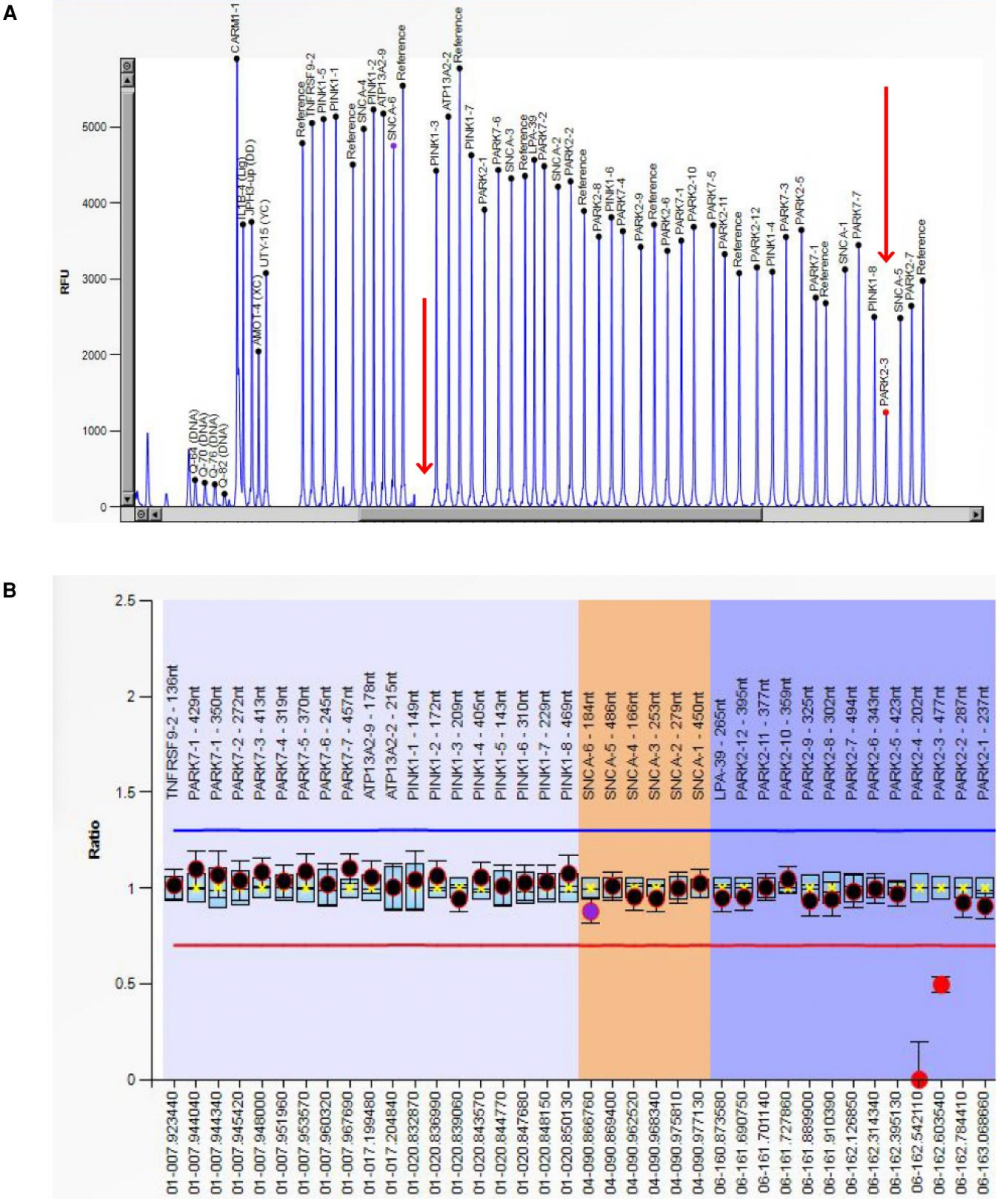
**Biallelic *PRKN* deletion identified in an early-onset Parkinson’s disease case.** (**A**) Multiplex ligation-dependent probe amplification (MLPA) was performed in early-onset Parkinson’s disease cases and/or individuals with positive family history. One early-onset Parkinson’s disease with an age at onset of 27 years was detected to have a decrease in the height of the electropherogram peak at 202 and 477 nucleotides (red arrows), which correspond to *PRKN*-4 and *PRKN*-3 probes, respectively. (**B**) The probe ratios indicate a *PRKN* exon 3 heterozygous deletion (0.5 ratio) and a *PRKN* exon 4 homozygous deletion (0 ratio).

We obtained the pathogenicity scores of the candidate variants from CADD, AlphaMissense and PrimateAI. A score >12.36 for CADD, > 0.564 for AlphaMissense and >0.803 for PrimateAI are recommended as pathogenic cut-offs. None of the previously reported pathogenic variants in known genes fulfilled the pathogenicity criteria for AlphaMissense and PrimateAI. Therefore, although we included scores from these tools, we did not apply any filtering based on the recommended thresholds. All identified variants in recessive PD genes were present in single heterozygous states (Figs 4 and 5 and Supplementary Table 3). A female case with PD from the PDGENE study with an AAO under 30 was identified as a carrier of two heterozygous variants in *PRKN* (p.Gly139Valfs*38 and p.Pro113Thrfs*51) that would suggest a potential compound heterozygous state (Table 2 and Supplementary Table 4). We were unable to confirm compound heterozygosity due to the absence of phasing data. The distribution of AAO among prioritized variant carriers in PD genes (*PRKN*, *DJ-1*, *PINK1*, *VPS13C*, *DNAJC6*, *GBA1*, *SNCA*, *LRRK2*, *TRPM7* and *VPS35*) is shown in Fig. 4.

#### Rare *GBA1* variant landscape differs African and African admixed ancestry patients

We identified a total of 18 heterozygous *GBA1* variants among 26 cases (4%), including 10 previously reported as pathogenic or likely pathogenic, four novel variants and four variants of uncertain clinical significance (Figs 2, 3, Tables 1 and 2 and Supplementary Fig. 2 and Supplementary Table 4). To identify potential known variants in the pseudogene paralogue *GBAP1* exons 9–11 homology region, we screened 15 whole-genome sequencing samples. The Gauchian algorithm predicted four carrier mutations, including variants p.Asp448His, c.1263del and c.1263del+Rec*TL*, i.e. deletion combined with pseudogene variant in exon 9 and three in exon 10. The algorithm predicted no structural variants: a total of four copies per *GBA1* and *GBAP1* variant in each sample were identified. Overall, four variants, including p.Gly416Ser, p.Trp223Arg, p.Arg170Leu and p.Thr75del in *GBA1* were targeted outside the homology region.

The previously reported pathogenic p.Arg170Leu (rs80356763) variant was identified in a family with two early-onset female PD siblings of African genetic ancestry, with ages at onset in their early 30s and 40s. The p.Thr75del (rs761621516) variant was identified in two cases from PDGENE and one case from the GP2-BLAAC PD dataset with no familial history. The carriers from the PDGENE were two Black or African/African American male patients (one with confirmed African genetic ancestry), who had onset in their 50s and early 70s. The carrier from the GP2-BLAAC PD dataset was a female patient with a disease onset in her late 50s. Both of these variants appear to be specific to African and African admixed ancestries, with no records in other ancestries according to gnomAD v4.1.0 and All of Us release 7 (Table 1 and Supplementary Table 3).

Four additional known pathogenic *GBA1* variants, including p.Arg535His (1:155235002:C:T, rs75822236), p.Asp448His (1:155235727:C:G, rs1064651), p.Arg502Cys (1:155235196:G:A, rs80356771) and p.Asn227Ser (1:155238215:T:C, rs364897), were identified in patients from the GP2-BLAAC PD release 8 data (Table 1). The p.Arg535His variant was identified in a female individual of African ancestry with disease onset in her 70s, whereas the p.Asp448His and p.Arg502Cys variants were identified in female individuals of African admixed ancestry with onset in their 50s. The p.Asn227Ser variant was identified in a male patient of African ancestry with onset in his early 50s.

Two previously reported pathogenic variants, p.Ala495Pro (1:155235217:C:G, rs368060) and p.Gly416Ser (1:155235823:C:T, rs121908311), were found in two female patients in the All of Us dataset (Table 1). Both patients were of African ancestry and diagnosed in their 50s. Additionally, the known missense p.Trp223Arg (1:155238228:A:G, rs61748906) variant was identified in a male patient of African genetic ancestry from the NPDRN cohort. He presented with initial symptoms in his early 60s and reported no family history. The stopgain p.Arg202Ter (1:155238291:G:A, rs1009850780) variant was found in a female patient from the PDGENE dataset, with onset in her late 50s (Table 1).

Four novel variants in *GBA1* were identified (Table 2). Of note, none of these were found to be present in the *GBA1*-PD browser (https://pdgenetics.shinyapps.io/gba1browser/, accessed 1 November 2024). The missense *GBA1* p.His413Arg variant (1:155235831:T:C, rs911331923, CADD = 23.9) was identified in a male case from the GP2-BLAAC PD cohort, with disease onset in his 40s. This variant is specific to African/African American ancestry, reported only in individuals of these ancestries in the gnomAD v4.1.0 (allele count = 3) and All of Us release 7 (allele count = 7) browsers, respectively. The histidine to proline change at this position was previously reported as likely pathogenic, whereas the p.His413Arg variant identified in the current study has not been reported before. The second novel missense *GBA1* p.Ile407Ser variant (1:155236249:A:C, rs1057519358, CADD = 26.5) was identified in a male patient of African admixed genetic ancestry from the GP2-BLAAC PD cohort with disease onset in his early 50s. While *GBA1* p.Ile407Thr was previously reported with an uncertain clinical significance, p.Ile407Ser has not been reported before. The third novel variant identified in this study was *GBA1* p.Gly364Arg (1:155236379:C:G, CADD = 20.2) in a male patient with slowness and/or minimal cognitive problems from the GP2-BLAAC PD dataset, who had his initial symptoms in his late 50s. While a cysteine to thymine substitution at this position was previously reported, this specific variant is novel and absent in gnomAD v4.1.0. Lastly, a frameshift variant, *GBA1* p.Ala300Profs*4 (1:155237441:GC:G, CADD = 28.7), was identified in an early-onset female case of African genetic ancestry from the GP2-BLAAC PD dataset, who presented with initial PD symptoms in her early 40s and had a second-degree relative affected by the disease.

The *GBA1* p.Met400Ile variant (1:155236269:C:T, rs149487315), previously classified as having uncertain clinical significance, was the most prevalent variant among the screened PD genes, with a carrier frequency of 0.84% across all cases and an allele frequency of 0.0028 in the gnomAD African populations. This variant is more prevalent in individuals of African ancestry, with an allele count rate of 91% in the All of Us dataset (release 7) (Supplementary Table 4).

Although the focus of this study was not to screen for common or non-coding variants, we screened the recently identified genetic risk factor linked to PD risk in the African and African American populations (rs3115534-G). Our study identified 3324 *GBA1-*rs3115534-G heterozygous (3207 in African and 117 in African admixed) and 320 homozygous carriers (311 in African and nine in African admixed), with a risk allele frequency of 0.23 in cases and 0.17 in controls in African ancestry and a frequency of 0.15 in cases and 0.14 in controls in African admixed individuals. The frequency of the G allele in each dataset tested is presented in Supplementary Table 5.

#### European and Asian *LRRK2* variants are not major risk factors in West African or African admixed patients

The current study did not identify any pathogenic or likely pathogenic *LRRK2* variants previously reported in Europeans (p.Asn1437His, p.Arg1441Ser, p.Arg1441Gly, p.Arg1441Cys, p.Arg1441His, p.Val1447Met, p.Tyr1699Cys, p.Phe1700Leu, p.Ile2020Leu and p.Ile2020Thr) or Asian ancestries (p.Gly2385Arg). Three novel heterozygous missense *LRRK2* variants of uncertain significance were found in the All of Us and PDGENE datasets (Table 2). A missense p.Glu268Ala substitution (12:40243646:A:C, rs373254349, CADD = 24.6) was identified in a female patient with disease onset in her early 60s. A p.Ile1438Lys variant (12:40309228:A:T, CADD = 24.2) was found in a PDGENE participant over 60 years old (age at onset was unavailable) with a family history of PD. The third missense variant, *LRRK2* p.Arg1538Cys (12:40314047:C:T, rs150620977, CADD = 29.2), was detected in a female patient from All of Us, with disease onset in her early 40s and a disease duration of 15 years. The *LRRK2* p.Glu268Ala and p.Arg1538Cys variants were more prevalent in individuals of African ancestry in the All of Us dataset (release 7), with allele count rates of 93.75% African (6.25% European) and 87.5% African (8.3% American, 1.40% European, 2.78% other), respectively, across the entire dataset. The *LRRK2* p.Ile1438Lys variant was absent in both gnomAD v4.1.0 and All of Us.

#### Novel variation in Parkinson’s disease genes warrants further replication and functional validation

A p.Met116Ile missense variant (4:89729236:C:A, rs1378041201, CADD = 18.9) in *SNCA* was identified in a male patient of African ancestry from the PDGENE dataset, with disease onset in his late 50s and no family history of PD (Supplementary Table 3). No clinical information about dementia was available. While a C to T transition at the same position was reported in a single individual of East Asian genetic ancestry in gnomAD, the variant was absent in gnomAD v4.1.0.

The *VPS35* missense variant of uncertain significance p.Met607Val (16:46662991:T, rs1555523076, CADD = 21.5) was identified in a male African ancestry PD patient in the NPDRN dataset, while the p.Asp205His variant (16:46679050:C, CADD = 29.4) was identified in a male patient of African admixed ancestry in the GP2-BLAAC PD (Supplementary Table 4). The individual carrying the p.Met607Val variant had disease onset in his early 40s and a positive family history, with a second-degree relative affected by the disease. At the time of enrolment, he had Hoehn and Yahr stage 2 and had demonstrated a sustained response to levodopa without dyskinesia. In terms of non-motor features, he showed no signs of hyposmia or cognitive impairment (MDS-UPDRS Part 1.1 cognition score of 0). Notably, the p.Met607Val variant is 13 amino acids from the known pathogenic p.Asp620Asn variant and lies in the C terminal domain. The patient carrying the p.Asp205His variant of uncertain significance presented with initial symptoms in his late 40s and reported no family history of PD. Using AlphaFold and PyMOL, we visualized the predicted structural consequences of the novel amino acid changes. This also includes estimates of protein stability, with more negative ΔΔG values generally indicating greater instability (Supplementary Fig. 4).

#### Integration of local ancestry analysis with variants of interest reveal population-specific variants

Local ancestry inference revealed that the majority of prioritized variants were carried on haplotypes of African ancestry (Tables 1 and 2 and Supplementary Figs 6 and 7). Specifically, several *GBA1* variants including p.Asp448His, p.Thr75del, p.Ala300Profs4, p.Arg170Leu, p.Arg535His, p.Asn227Ser, p.Gly364Arg, p.Trp223Arg and p.His413Arg were all found on African ancestry haplotypes. Additional variants of African ancestry origin included *VPS35* p.Asp205His and p.Met607Val, as well as *SNCA* p.Met116Ile. One variant (*PRKN* p.Asn52Metfs29) was found on a South Asian ancestry haplotype, and *GBA1* p.Arg502Cys was assigned to a European ancestry background. For *GBA1* p.Ile407Ser, the local ancestry could not be confidently resolved to either African or European origin. A stopgain variant, *FBXO7* p.Arg321Ter, and the missense variant *PLA2G6* p.Thr319Met were located on haplotypes of Middle Eastern and African ancestry, respectively. Ancestry inference for the *PRKN* p.Pro113Thrfs*51 deletion was not possible.

#### *PRKN* copy number variants linked to Parkinson’s disease in African and African admixed populations

We screened 96 individuals for CNVs using MLPA. We prioritized samples by individuals positive for both family history and early-onset PD (*n* = 4), only family history (*n* = 15), only early onset PD (*n* = 36) and randomly selected sporadic PD cases (*n* = 41). We identified one early-onset PD case from the GP2-BLAAC PD dataset having a *PRKN* exon 3 heterozygous deletion and a *PRKN* exon 4 homozygous deletion (Fig. 5). This PD case had an AAO of 27 years and no family history of PD. Mimicking the *PRKN*-PD clinical phenotype previously reported, this individual was levodopa responsive, and was positive for asymmetric onset, bradykinesia, rest tremor, rigidity and gait difficulties. In our additional screening of 1167 PD cases of African and African admixed ancestry using genotyping data, we identified *PRKN* CNVs in eight samples (African admixed = 5, African = 3), two of which were homozygous (African admixed = 1, African = 1). Out of these, five were early-onset cases (Supplementary Table 6 and Supplementary Fig. 3). We did not identify any protein-altering or splicing *PRKN* variant among these samples. We did not identify any CNVs in *SNCA*.

#### Previously reported pathogenic variants in parkinsonism and neurodegenerative disease-related genes

We identified heterozygous variants in genes associated with recessive PD and parkinsonism, including one in *DNAJC6*, six in *VPS13C*, two in *SYNJ1*, three in *PLA2G6* and one in *FBXO7* in early-onset cases. Among these, the homozygous *FBXO7* p.Arg321Ter variant (22:32491175:C:T, rs369105683) was reported as pathogenic in ClinVar. However, this variant was found to be heterozygous in the current study. We also reported rare heterozygous variants in neurodegenerative disease-related genes, including *TRPM7*, *MAPT* and *APP* that could be associated with parkinsonism phenotypes; however, none have been previously reported as pathogenic. Notably, we identified a homozygous p.Ala128Gly variant (17:45982962:C:G, rs899291077) in *MAPT* in a male case from the GP2-BLAAC PD dataset. Variants in *MAPT* were previously reported in cases with progressive supranuclear palsy, an atypical parkinsonian and dementia syndrome.^29^ The carrier of this variant in our study presented with resting tremor, bradykinesia and rigidity in his 70s, with no evidence of dementia (Supplementary Table 3). None of the cases with *APP* variants exhibited dementia or cognitive impairment. We identified rare variants in parkinsonism and dystonia genes, including novel *VPS13D* homozygous variants in four cases, and heterozygous variants in *PD8EB*, *KCNN2*, *CSF1R*, *THAP1*, *TOR1A*, *ANO3*, *GCH1*, *VPS16*, *KCDT17*, *KMT2B* and *ATP1A3* genes. These variants were novel or previously reported with unknown clinical significance (Supplementary Table 7).

#### Runs of homozygosity highlight regions overlapping Parkinson’s disease and atypical parkinsonism

In the African and African admixed groups, 164 and 43 ROH pools, respectively, were identified, which overlap known PD and atypical parkinsonism gene regions and GWAS loci, respectively (Supplementary Tables 8 and 9). None of the ROH regions enriched in cases for either group passed the Bonferroni correction. However, in the African group, one ROH pool (S2421) that overlaps the *BAG3* and *INPP5F* genes was present in four cases and completely absent in controls. Moreover, in the African admixed group, two ROH pools (S622 and S636) were found in cases and absent in controls. These pools overlap the *MEX3C* (*n* = 2 cases), *SMAD4* (*n* = 2 cases) and *PLA2G6* (*n* = 2 cases) genes (Supplementary Tables 8 and 9).

#### Spinocerebellar ataxia type 3 associated repeat expansions in African and African admixed cases

*In silico* analysis using ExpansionHunter identified *ATXN3* repeat expansions within the pathogenic range (CAG_n_ > 45) in three PD patients (1.02%) of African ancestry (two cases from the GP2-BLAAC PD dataset and one case from the NPDRN cohort) and in one control (0.34%) from the NPDRN cohort. Repeat-primed PCR (RP-PCR) validation confirmed the expansions in two cases and one control, with repeat lengths of (CAG_n_ = 72, CAG_n_ = 58 and CAG_n_ = 52, respectively). The carrier of the CAG_n_ = 72 expansion is a female patient of African ancestry with an AAO of 41 years. The carrier of the CAG_n_ = 58 expansion is also a female patient with an AAO of 35 years. The control carrier, with CAG_n_ = 52 is a 40-year-old male. Owing to the lack of a biospecimen, we did not perform RP-PCR for the fourth potential carrier, an African ancestry male patient from the NPDRN cohort with an AAO of 35 years, identified through *in silico* analysis. However, visualization of wild-type and expanded reads from short-read sequencing data is presented in Supplementary Fig. 5. None of the *ATXN3* expansion carriers were found to carry any additional rare variants in known PD genes.

STR lengths in the *ATXN2* and *TBP* were within the normal range. The lengths of STRs in *ATXN2*, *ATXN3* and *TBP* for all individuals from the GP2-BLAAC PD and NPDRN datasets are presented in Supplementary Table 10.

## Discussion

We undertook the largest sequencing characterization of PD, parkinsonism and neurodegenerative disease-related genes in the African and African admixed populations. We created the most comprehensive catalogue of both known and novel coding and splicing variants potentially linked to PD in these populations. Notably, our local ancestry analysis revealed that the majority of prioritized variants were enriched on African-derived haplotypes, since African populations harbour the greatest genetic diversity, and regions of the genome with African-derived haplotypes are expected to show single nucleotide polymorphism enrichment compared with non-African segments. Local-ancestry analyses that lump all African haplotypes together can miss substructure-specific differences (e.g. a variant common in West African populations but rare in East or Southern Africans), which constrains our ability to resolve subcontinental structure. While our study was not powered to systematically compare African and African admixed individuals, these remain important considerations because haplotype background can influence variant interpretation, pathogenicity, allele frequency distributions and effect size estimates. For African and African admixed populations, this framework is especially critical given the high genetic diversity and complex admixture histories. Admixture results for the GP2-BLAAC PD, NPDRN and PDGENE samples are presented in Supplementary Fig. 6 and Supplementary Tables 11 and 12.

Biallelic *GBA1* variants are associated with Gaucher’s disease, while monoallelic *GBA1* variants are the most prevalent genetic risk factor for PD, with prevalence varying among diverse ethnic groups.^30-35^ We identified that heterozygous rare and potentially disease-causing *GBA1* variants represent the most commonly identified mutations in PD among patients of African and African admixed ancestry, with a frequency of 4%, aligning with the frequency of 4.6% reported in North African populations.^36^ Our study identified 18 heterozygous *GBA1* variants, five of which appear more frequent in African ancestry than other ancestry populations according to gnomAD v4.1 and All of Us (release 7), including p.His413Arg, p.Met400Ile, p.Asn227Ser, p.Ser77Arg and p.Thr75del. Of those, p.Asn227Ser was classified as severe, p.Thr75del as mild, p.Met400Ile as unknown, while p.His413Arg and p.Ser77Arg variants have not been previously reported.^37^ No carriers were identified for the most common disease-causing *GBA1* variants in the Ashkenazi Jewish and European populations (p.Asn409Ser, p.Leu483Pro, p.Thr408Met and p.Glu365Lys).

The recent PD GWAS in the African and African admixed ancestry populations revealed a non-coding variant within *GBA1* (rs3115534-G) conferring a major population attributable risk in patients.^6^ Follow-up functional characterization highlighted a novel mechanism of *GBA1*-related PD risk, offering a promising target for precision medicine in a population that has been historically underserved in genetic research and therapeutic development.^7^ Though this study was primarily focused on assessing the impact of coding variants, we specifically screened for this variant in our cohort. Our analyses showed a higher estimate of the risk G allele in predicted African ancestry individuals, with an overall frequency of 0.23 in cases and 0.17 in controls and a frequency of 0.15 in cases and 0.14 in controls among individuals of African admixed ancestry.

Our results showed that the previously reported European and Asian *LRRK2* mutation spectrum does not play a significant role in PD in West African and African American populations. For instance, the *LRRK2* p.Gly2019Ser variant was absent in tested cases. It is known that *LRRK2* p.Gly2019Ser shows varying frequencies across North African genetic ancestry groups, ranging from 10% to 42%, with the highest rates reported in Tunisia.^38-44^ Conversely, this variant is rare among sub-Saharan Africans, with only a few cases reported in South Africa.^45^ A study screening for p.Gly2019Ser in 109 Nigerian patients did not identify any variant carriers.^46^ Additionally, *LRRK2* p.Gly2019Ser carriers were not detected among 16 South African patients^47^ and 38 Zambian individuals with PD.^48^ Our results align with a recent study examining *LRRK2* mutations in 22 African American PD patients.^3^ Although the authors identified both known and novel variants, no previously reported pathogenic mutations were detected, in contrast to what has been observed in North African populations. Interestingly, we identified a heterozygous *LRRK2* p.Gly8Arg variant, a novel coding mutation not previously reported in the literature, considered to be of uncertain significance, present only in populations of African and admixed American ancestry according to gnomAD v4.1 and All of Us browsers (release 7). Similarly, the *LRRK2* p.Glu1797Asp heterozygous variant was identified in our study among two male PD cases of African ancestry; this variant has been shown to be present in African and admixed American ancestry populations and absent in Europeans (73% African and 23% admixed American ancestry in All of Us release 7). Overall, these findings highlight ancestral differences in *LRRK2* variant distribution, underscoring the need for further research to explore these disparities. The discovery of novel variants opens new avenues for functional investigations.

Among the novel variants identified, *GBA1* p.Ile407Ser and *LRRK2* p.Ile1438Lys were predicted to cause greater protein instability (Supplementary Fig. 4). The *GBA1* p.Ile407Ser showed the strongest destabilization (ΔΔG = −2.98), which may suggest a potential dual mechanism. First, reduced glucocerebrosidase activity suggests a potential loss-of-function mechanism, consistent with pathogenic variants in the same region such as p.Asn409Ser and p.Leu444Pro, which are known to reduce β-glucocerebrosidase activity and increase levels of glucosylceramide and α-synuclein.^49^ However, it is worth noting that several studies support the general hypothesis (not variant specific), in which impaired trafficking may lead to accumulation of aberrant glucocerebrosidase species and proteostatic stress, implying a toxic gain-of-function component.^50^ The *LRRK2* p.Ile1438Lys variant (ΔΔG = −1.72) lies in the ROC domain near the p.Arg1441His mutational hotspot, where destabilization is thought to enhance kinase activity of *LRRK2,* supporting a gain-of-function mechanism.^51^

Our study aligns with previous research showing that *SNCA* coding and structural variation is rare in African ancestry populations.^52^ Our analysis did not detect any coding variants or structural rearrangements in *SNCA* across the cohorts studied. This finding is consistent with several other studies on PD in African and African admixed populations. For example, a study of 38 PD cases from Zambia (plus one with atypical parkinsonism) found no pathogenic *SNCA* mutations.^48^ Similarly, a screening of 15 early-onset PD cases from southwestern Nigeria,^46^ and a study of 202 South African PD cases (8% Black) also found no exonic *SNCA* variants linked to disease.^53^

Regarding recessive genes, nearly 20 PD cases associated with *PRKN* and *PINK1* mutations have been documented in sub-Saharan Africa across multiple studies.^48,52,54-58^ In contrast, fewer than 10 PD cases with these mutations have been reported in North Africa.^44,59-64^ Though compound heterozygous *PRKN* mutations cause recessive disease, heterozygous protein altering variants do not appear to confer PD risk, with multiple null associations reported.^65-67^ In our study, we identified two heterozygous *PRKN* variants in an early-onset PD case, one of which had been previously reported as pathogenic, suggesting a potential compound heterozygous state that was not confirmed due to the lack of phasing data. Together with the CNVs identified in eight cases—three from African and five from African admixed ancestry —a total of 14 early-onset PD cases were found to carry potential disease-causing *PRKN* mutations (Tables 1 and 2 and Supplementary Table 4).

*PRKN* carriers constituted 1.3% (9 of 710) of all cases. *PRKN* variants constituted 13.6% of all observed variants in PD genes, with 13 variants in the GP2-BLAAC PD cohort and 10 in the NPDRN. Of those, nine variants were more frequent in cases than controls and seven were more frequent in African ancestry individuals, relative to non-Finnish European ancestry, according to gnomAD. We identified two heterozygous *PINK1* variants, previously reported as pathogenic, in two late-onset PD cases, which were likely not the cause of underlying PD in these cases. While we did not report any pathogenic variants in *DJ-1* and *VPS13C*, we found four novel *DJ-1* and 13 novel *VPS13C* variants, all in heterozygous state, that warrant further study.

We identified SCA3-associated *ATXN3* repeat expansions in three PD patients and one African ancestry control. Although the control individual is currently unaffected, the presence of a pathogenic-range expansion raises the possibility of future disease manifestation, highlighting the importance of continued follow-up. Notably, a similar analysis of a large European ancestry PD cohort from the Accelerating Medicines Partnership Program for Parkinson’s Disease did not identify any *ATXN3* expansion carriers (personal communication, unpublished data). Previously, Gwinn-Hardy *et al*.^18^ reported PD-like parkinsonism in African American individuals carrying pathogenic *ATXN3* repeat expansions. In their study, three of four affected family members exhibited classic parkinsonian features and were responsive to levodopa.^18^ Our findings further support the possibility that *ATXN3* expansions may contribute to early-onset or atypical parkinsonism in individuals of African and African admixed ancestry and highlight the importance of incorporating STR analysis into PD genetic studies in diverse populations. Screening for *ATXN3* repeat expansions may be especially informative in parkinsonian cases lacking known pathogenic variants.

Several limitations should be acknowledged. First, our study includes a highly heterogeneous population. The current ancestry reference panels cannot accurately distinguish African subpopulations. African admixed study participants commonly exhibit diverse heritage encompassing African, European, and in certain instances, Native American genetic components. The African component of African American ancestry is itself varied, reflecting the multitude of ethnic groups involved in the trans-Atlantic slave trade. Most of the PD studies encompassing individuals of African ancestry primarily stem from West African populations, which capture only part of Africa’s genetic diversity and do not represent the continent in its entirety.^68^ We acknowledge the limitation that ancestry inferences were estimated under the assumption that the reference panel consists of non-admixed individuals. In our study, the reference panel was constructed from GP2 ancestry clusters, which, while broadly representative, may still include individuals with subtle admixture, potentially introducing uncertainty in ancestry classification. Future pipelines incorporating larger and more diverse reference panels, as well as probabilistic ancestry deconvolution approaches, will improve our ability to infer local ancestry with greater precision. Second, most candidate variants identified in our study are singletons, observed only in a single individual or within a single dataset. Therefore, caution is warranted in interpreting their causality. In addition, variants across the *GBA1* and *GBAP1* homology region require further analysis for validation using other tools, since Gauchian cannot detect novel variants in this region. Third, structural variations were not comprehensively captured due to limitations in both detection strategy and sequencing technology. Our CNV analysis was restricted to *SNCA* and *PRKN*, two genes where CNVs were previously reported in PD. However, most samples did not undergo MPLA, reducing sensitivity and confidence of CNV detection and precluding formal association testing. Long-read technologies are more effective in detecting large insertions or deletions, which may not be captured by short-read sequencing or MLPA. Incorporating long-read sequencing in future studies could enhance our understanding of the genetic underpinnings of PD in these populations. Furthermore, since the data were being hosted on different platforms, joint calling was not possible. We therefore did not merge the sequencing datasets. Given these limitations in sample composition and cohort harmonization, we currently lack sufficient statistical power to conduct robust single-variant association or gene-based burden testing. Clinical information was also lacking; only a subset of samples had data from the Montreal Cognitive Assessment (MoCA) and few had data for the Identification and Intervention for Dementia in Elderly Africans (IDEA) cognitive screen and REM sleep behaviour disorder (RBD). One question from the CISI-PD questionnaire was used to assess cognitive status. Future data collection efforts as part of the GP2-BLAAC PD study will include the MoCA and other clinical assessments.

Our study, the largest of its kind to explore rare coding and splicing variations in African and African admixed populations, aims to fill critical gaps in PD genetics by providing a comprehensive genetic characterization of these ancestries. While recognition of the importance of studying PD in diverse populations is growing, Black and African admixed communities have long been underrepresented in research, creating major gaps in understanding how PD manifests and progresses. By focusing on these underserved groups, our work seeks to expand knowledge of PD aetiology, ultimately improving diagnostic accuracy and therapeutic development. As we advance toward precision medicine, therapies should be developed with a global perspective. Operating under the umbrella of the GP2, it is essential that data guiding individualized treatments reflects population diversity, ensuring equitable benefits from medical advancements in PD care.

## Supplementary Material

awaf379_Supplementary_Data

## Data Availability

All GP2 data for these analyses is available through collaboration with Accelerating Medicines Partnership in Parkinson’s disease (AMP-PD) initiative . The GP2-BLAAC PD, NPDRN, and PDGENE datasets are all accessible via GP2 (release version 7 and version 8), available through application on the AMP-PD platform (https://amp-pd.org/register-for-amp-pd). Other publicly available data consortiums include All of Us Research Program (https://www.researchallofus.org/register/) and UK Biobank (https://www.ukbiobank.ac.uk/enable-your-research/register), in which data can be accessed after applying. We have received an exception to the Data and Statistics Dissemination Policy from the All of Us Resource Access Board. Analyses such as genotyping quality control, ancestry prediction and processing were performed using GenoTools (https://github.com/dvitale199/GenoTools). A repository containing all code used for processing and analysing is publicly available (https://github.com/GP2code/PD-GeneticCharacterization-inAFRandAAC/; DOI 10.5281/zenodo.14579574).
