# Supplementary material for: Large-scale genetic characterization of Parkinson’s disease in the African and African admixed populations
Source: Brain. 2025 Oct 8;149(5):1537–53. doi: 10.1093/brain/awaf379 (PMC13140531; doi:10.1093/brain/awaf379)
Supplement: awaf379_Supplementary_Data [file awaf379_supplementary_data.zip › brain-2025-00177-File009.pdf]

Supplementary Methods

Sequencing procedures

GP2-BLAAC PD and NPDRN

Short-read whole genome sequencing (WGS) was performed by Psomagen (Rockville, MD, USA) using Illumina's TruSeq DNA PCR-Free High Throughput Library Prep Kit (cat. # 20015963, Illumina, San Diego, CA, USA) [1]. Sequencing was performed in 277 individuals from GP2-BLAAC PD and 168 individuals from the NPDRN (108 PD cases and 60 controls).

The functional equivalence pipeline [2] from the Broad Institute was used to produce alignments and call small variants against a reference genome (GRCh38). We followed the quality metrics defined by the Accelerating Medicines Partnership Parkinson's Disease initiative (AMP-PD; https://amp-pd.org) [3] for sample-level WGS quality control. Joint-genotyped variants for all samples that passed quality control were generated from the Broad Institute's joint discovery pipeline. Only the high-quality variants flagged as "PASS" following variant quality score recalibration, with call rate >0.95, genotype quality >=20, read depth >=5, and heterozygous allele balance between 0.25 and 0.75 were retained, as described previously [4].

In addition to WGS, targeted sequencing was performed at Uniformed Services University of the Health Sciences (USUHS) in 383 additional GP2-BLAAC PD samples including 149 cases and 234 controls utilizing the Illumina TruSight One Sequencing Panel, which includes 4,800 genes and 12 Mb of genomic content (cat. 20025524 & 20029227, Illumina, San Diego, CA, USA). DNA samples were normalized to 50ng and quality control was performed with Qubit 4 Fluorometer. Seven samples did not pass quality control and were discontinued; 376 total samples were included for sequencing. Index, paired-end libraries at 150bp were produced using Illumina DNAPprep with Enrichment Tagmentation (cat # 20025524, Illumina, San Diego, CA, USA) and unique dual index sets (IDT for Illumina DNA/RNA UD Indexes Sets A, B, C, and D Tagmentation). Samples were pooled in batches of 96 samples and sequenced on the Illumina Novaseq6000. The sequencing data processing followed the same pipeline of WGS data as mentioned above. We performed joint-genotyping using Broad Institute's joint discovery pipeline and followed the same criteria for sample and variant quality control as for the WGS data.

PDGENE

Targeted next-generation sequencing data were utilized from the PDGENE dataset in GP2 release 8. Sequencing was performed by Fulgent Genetics, designed to examine coding regions and splicing junctions (https://www.fulgentgenetics.com/parkinson-disease-comprehensive). Details regarding the sequencing procedure for this dataset and data curation were described in Cook et al. 2024 [5].

All of Us

Participants were included from All of Us Controlled Tier Dataset release 7. Sequencing was performed by the Genome Centers funded by the All of Us Research Program [6,7]. All genome centers used the same sequencing

protocols that consisted of PCR-free 150 bp, paired-end libraries sequenced on the Illumina NovaSeq 6000 platform. Sequencing data was processed using DRAGEN v3.4.12 (Illumina, San Diego, CA, USA). The GRCh38 reference genome was used for alignment [8].

UK Biobank

Further details on whole-exome sequencing, variant calling, and quality control protocols can be found at Van Hout et al. 2020 and Backman et al. 2021 [9,10]. In brief, samples were transferred to the Regeneron Genomics Center, where DNA libraries were created with mean sheared DNA fragment sizes of 200bp and unique asymmetric 10bp barcodeswere added. Exomecapturewas performed using a variation of the IDT xGen Exome Research Panel v1.0, with two different lots being used between the first 50,000 samples and all other samples. DNA was amplified and quantified using qPCR before being sequenced using the Illumina NovaSeq 6000 platform with 75bp paired-end reads. S2 flow cells were used for the first 50,000 samples, while S4 flow cells were used for all remaining samples. Raw data were mapped to the GRCh38 reference genome using BWA-mem. Individual-level gVCF files were generated using the WeCall variant caller, which were joint-called into project-level pVCF files using GLnexus.

The rs3115534 intronic variant was obtained from UK Biobank's whole-genome sequencing datasets. Whole-genome sequencing was conducted using the NovaSeq 6000 platform (https://www.medrxiv.org/content/10.1101/2023.12.06.23299426v1). These data were then analyzed with the DRAGEN v3.7.8 (Illumina, San Diego, CA, USA) software. Alignment was performed against the GRCh38 reference genome. Further details on quality control metrics can be found at https://biobank.ndph.ox.ac.uk/showcase/label.cgi?id=187.

Genotyping, quality-control and genetic ancestry estimation

The GP2-BLAAC PD samples were genotyped using the NeuroBooster (version 1.0; Illumina, San Diego, CA, USA) array platform [11]. The array included 1,914,935 variants that encompass ancestry markers, identity by descent markers, and biological sex markers derived from X-chromosome single-nucleotide polymorphisms (SNPs). Out of these variants, 96,517 variants were customized based on neurodegenerative disease. The NPDRN samples were genotyped on both the NeuroBooster and the NeuroChip arrays (version 1.0; Illumina).

Genotyping data generation, quality control, and ancestry predictions were conducted as follows: The machine learning method from the GenoTools pipeline (https://github.com/GP2code/GenoTools) was used to perform custom ancestry prediction and quality control on genotyping data according to previously described methods in Vitale et al. 2024 [12]. Samples that failed quality control criteria were excluded based on call rate (<95%), sex determination inconsistency from the clinical data, or excess heterozygosity detection (|F| statistics > 0.15). Variants with Hardy-Weinberg Equilibrium $P \leq 1 \times 10^{-4}$ in control samples were removed after the preliminary sample-level quality control was completed. Variants were further pruned to remove variants with non-random missingness first by using a cutoff of case-control status at $P \leq 1 \times 10^{-4}$ and then by haplotype at $P \leq 1 \times 10^{-4}$.

Predicted change in protein structures

The predicted protein structures encoded by eight genes associated with PD (*GBA1*, *LRRK2*, *DJ-1, PINK1*, *PRKN*, *SNCA*, *VPS13C*, *VPS35*) were obtained from the EMBL AlphaFold Protein Structure Database (accessed May, 2025) to ensure that all of the residues are present in each protein structure [13,14]. The structural consequences of missense and other coding variants were evaluated using DynaMut2, a computational tool that integrates normal mode analysis and graph-based signatures to predict the impact of mutations on protein stability and flexibility [15]. This approach allowed for the estimation of ΔΔG (change in folding free energy) and alterations in vibrational entropy upon mutation, providing insight into potential functional disruptions at the structural level. All protein structures and mapped mutation sites were visualized using PyMOL v2.6.0 (The PyMOL Molecular Graphics System, Version 2.6.0; Schrödinger, LLC).

Ancestry inference

Ancestry estimates for the GP2-BLAAC PD, the NPDRN, PDGENE, and UKB datasets were conducted by GenoTools[12] (https://github.com/GP2code/GenoTools) using reference panels from the 1000 Genomes Project [16], Human Genome Diversity Project [17], and an Ashkenazi Jewish population [18]. A standard protocol across all samples was used to perform ancestry estimates. Briefly, the referencepanel was randomly divided into training (80%) and testing (20%) subsets. Principal components (PCs) were calculated using SNPs overlapping between the reference panel SNP set and the samples of interest and subsequently transformed using UMAP to capture both global genetic population structure and stochastic variation. A XGBoost classifier was trained on the UMAP-transformed PCs. In the test subset and through 5-fold cross-validation, the classifier consistently achieved balanced accuracies exceeding 0.95. This trained model was then applied to the whole dataset to infer ancestry for all samples.

Using the reference panel samples, ADMIXTURE (v1.3.0; https://dalexander.github.io/admixture/binaries/admixture_linux-1.3.0.tar.gz) was run in supervised mode. Under this model, the reference panel samples were assumed to be 100% of their assigned ancestry, allowing ADMIXTURE to estimate proportional ancestry contributions of the AFR, AAC, and other ancestry groups for the GP2-BLAAC PD, the NPDRN, and PDGENE samples. For detailsof thecloud-based and scalable genotype calling, quality control and ancestry estimation pipeline (model training, cross-validation and reference panel variant inclusion criteria), please see the GenoTools Github repository (https://github.com/GP2code/GenoTools). Estimated subpopulation ancestries for the GP2 -BLAAC PD, the NPDRN, and the PDGENE samples are provided in Supplementary Tables 11 -12.

Local ancestry analysis

Reference files were created using participants obtained from the 1000 Genomes Project Phase III [16]. Population substructure at a global level was investigated using ADMIXTURE v1.3.0[19] and visualized using the PONG software v1.5[20]. The genotyped files were phased using the TOPMed Imputation Server[21].

Finally, local ancestry was inferred using G-Nomix v1.0[21,22]. For each variant of interest, the corresponding local ancestry window was extracted from the G-Nomix output based on genomic coordinates. Variant positions were cross-referenced with the phased VCF file to identify the haplotype (strand) carrying the alternate allele. This haplotype was then compared to the inferred ancestry assignment within the overlapping local ancestry window to determine the ancestry background on which the variant resides. For variants not present in the imputed data, WGS data was phased with WhatsHap [23] and heterozygous variants in common between the genotyped and sequenced data were extracted. Local ancestry phase was established by aligning these and identifying the haplotype carrying the alternate allele.

In All of Us, principal components were generated from high-quality variants and projected onto HGDP and 1000 Genomes Project samples to estimate genetic ancestry using the Rye software as previously described in Conley et al. 2023 [24]. While both African and African admixed reference panels were included in the ancestry estimation for the GP2-BLAAC PD, NPDRN, PDGENE initiative and UKB, only the African reference panel was implemented for All of Us ancestry estimation. Ancestry inferences were applied to the 138 samples in the PDGENE dataset with available genotyping data while the remaining 145 samples wereincluded based on their self-reported Black or African American race.

Pairwise relatedness inferencewas estimated using the KING v.2.3.1 (Kinship-based INference for GWAS) software [25]. In brief, samples with a Kinship coefficient of at least 0.0884 were further explored for relatedness. Related samples are inferred based on the range of estimated kinship coefficients: >0.354, 0.354-0.177, 0.177-0.0884, and 0.0884-0.0442 that correspond to duplicate/monozygotic twin, 1st-degree, 2nd-degree, and 3rd-degree relationships, respectively.

Runs of homozygosity

To investigate genome-wide homozygosity, runs of homozygosity (ROHs) were identified using PLINK v.1.9 [26], as described below. A sliding window of 50 SNPs (--homozyg-window-snp) and 1,500kb in length (--homozyg-kb) was used in a stepwise approach as previously described [27]. A minimum of 100 SNPs (--homozyg-snp) was allowed with a threshold of one heterozygous SNP (--homozyg-window-het) and five missing SNPs (--homozyg-window-missing) in a ROH region. To enable the detection of shorter ROHs, a ROH region was called if each SNP covered a minimum of 5% of the homozygous sliding window (--homozyg-window-threshold). The ROH cut-off was set at >1.5Mb (--homozyg-kb), with a maximum allowable distance of 1Mb between consecutive SNPs (--homozyg-gap) and a minimum density requirement of one SNP per 50kb of the genome (--homozyg-density). Furthermore, we investigated ROH intersecting with known PD genes and GWAS loci as well as parkinsonism gene regions. Here, we used an approximate 1MB window both upstream and downstream from the known genes and GWAS hits [1,28–31].

Copy number variant detection

CNV detection was performed leveraging GP2-BLAAC PD genotyping data by assessing B allele frequency (BAF) and Log2 Ratio (L2R) estimates. Ninety-six cases from the GP2–BLAAC PD cohort were selected for CNV screening through a Multiplex Ligation-dependent Probe Amplification (MLPA) assay

using the SALSA® MLPA® reagent kit (#EK1-FAM) and SALSA® MLPA® Probemix P051 (#P051-100R), according to the manufacturer's instructions (MRC-Holland, Amsterdam, The Netherlands). Cases were prioritized for MLPA screening by family history of PD or an early age of onset (<50 years). Quantity of DNA was determined with a Qubit fluorometric dsDNA BR assay (#Q33326, Invitrogen, USA). DNA samples were diluted to 12.5 ng/uL in water for a total of 50 ng of DNA per sample and 1 µl of 50 mM Tris-HCl buffer at pH 8.5 (#BU-124S-85, Jena Biosciences, Germany) per reaction. PCR fragments were analyzed by capillary electrophoresis in an ABI 3730XL genetic analyzer (Applied Biosystems™, USA) using highly deionized (HiDi) formamide (#4311320, Applied Biosystems™, USA) and GeneScan™ 500 LIZ™ dye size standard (#4322682, Applied Biosystems™, USA). Data was analyzed using the Coffalyser.Net™ software package (MRC-Holland, Amsterdam, The Netherlands), according to the provided protocol.

Briefly, L2R ratio gives an indirect measure of the copy number of each SNP by plotting the ratio of observed to expected hybridization intensity. An R value above 1 is indicative of an increasein copy number (duplication or triplication), and an R valuebelow 1 suggestsa decrease (deletion). BAF plots the proportion of times an allele is called A or B at each genotype: thus the expected ratios are 1.0 (B/B), 0.5 (A/B) and 0.0 (A/A). Significant deviations from these figures in contiguous SNPs are indicative of a CNV. While this metric exhibits a high level of variance for individual SNPs, it does provide a measure of CNV when L2R values for numerous adjacent SNPs are visualized as described elsewhere [32]. BAF and L2R were extracted for *SNCA* and *PRKN* with ± 250 kb proximity of the target genes as these genes are particularly enriched for genetic rearrangements in PD.

Short tandem repeat expansion analysis

Lengths of the short tandem repeat expansions in *ATXN2*, *ATXN3*, and *TBP* were estimated using ExpansionHunter v5.0.0[33] and its published variant catalog, utilizing available alignment files from the GP2-BLAAC PD dataset and the NPDRN cohort. Potential disease-associated STRs were subsequently validated through repeat-primed PCR (RP-PCR) and fragment length analysis performed in Labcorp (https://www.labcorp.com/tests/620123/). To visualize the expanded CAG repeats in *ATXN3*, we used the REViewer tool (https://github.com/Illumina/REViewer).

Power calculation

Our study combines data from five cohorts generated through different sequencing platforms and methodologies. Notably, the largest number of cases comes from the PDGENE dataset, which lacks control samples, and the second-largest case cohort (number of cases = 186) comes from the GP2-BLAAC PD dataset. To evaluate the ability of our dataset to detect associations with rare variants, we performed power calculations based on the sample size of the GP2-BLAAC PD dataset using the online GAS Power Calculator (https://csg.sph.umich.edu/abecasis/gas_power_calculator/). For a variant with a disease allele frequency of 0.01 and a relative risk of 2, assuming a disease prevalence of 0.5%[28], the expected power to detect an association is 0.21. The power decreases to 0.1 for a relative risk of 1.5.

REFERENCES

1. Rizig M, Bandres-Ciga S, Makarious MB, et al. Identification of genetic risk loci and causal insights associated with Parkinson's disease in African and African admixed populations: a genome-wide association study. *Lancet Neurol*. 2023;22(11):1015-1025.

2. Regier AA, Farjoun Y, Larson DE, et al. Functional equivalence of genome sequencing analysis pipelines enables harmonized variant calling across human genetics projects. *Nat Commun*. 2018;9(1):4038.

3. Iwaki H, Leonard HL, Makarious MB, et al. Accelerating Medicines Partnership: Parkinson's Disease. Genetic Resource. *Mov Disord*. 2021;36(8):1795-1804.

4. Lange LM, Avenali M, Ellis M, et al. Elucidating causative gene variants in hereditary Parkinson's disease in the Global Parkinson's Genetics Program (GP2). *NPJ Parkinsons Dis*. 2023;9(1):100.

5. Cook L, Verbrugge J, Schwantes-An TH, et al. Parkinson's disease variant detection and disclosure: PD GENEration, a North American study. *Brain*. 2024;147(8):2668-2679.

6. All of Us Research Program Investigators, Denny JC, Rutter JL, et al. The "All of Us" Research Program. *N Engl J Med*. 2019;381(7):668-676.

7. Harrison SM, Austin-Tse CA, Kim S, et al. Harmonizing variant classification for return of results in the All of Us Research Program. *Hum Mutat*. 2022;43(8):1114-1121.

8. Venner E, Muzny D, Smith JD, et al. Whole-genome sequencing as an investigational device for return of hereditary disease risk and pharmacogenomic results as part of the All of Us Research Program. *Genome Med*. 2022;14(1):34.

9. Van Hout CV, Tachmazidou I, Backman JD, et al. Exome sequencing and characterization of 49,960 individuals in the UK Biobank. *Nature*. 2020;586(7831):749-756.

10. Backman JD, Li AH, Marcketta A, et al. Exome sequencing and analysis of 454,787 UK Biobank participants. *Nature*. 2021;599(7886):628-634.

11. Bandres-Ciga S, Faghri F, Majounie E, et al. NeuroBooster array: A genome-wide genotyping platform to study neurological disorders across diverse populations. *Mov Disord*. Published online September 16, 2024. doi:10.1002/mds.29902

12. Vitale D, Koretsky M, Kuznetsov N, et al. GenoTools: An open-source Python package for efficient genotype data quality control and analysis. *bioRxiv*. Published online March 29, 2024. doi:10.1101/2024.03.26.586362

13. Jumper J, Evans R, Pritzel A, et al. Highly accurate protein structure prediction with AlphaFold. *Nature*. 2021;596(7873):583-589.

14. Varadi M, Bertoni D, Magana P, et al. AlphaFold Protein Structure Database in 2024: providing structure coverage for over 214 million protein sequences. *Nucleic Acids Res*. 2024;52(D1):D368-D375.

15. Rodrigues CHM, Pires DEV, Ascher DB. DynaMut2: Assessing changes in stability and flexibility upon single and multiple point missense mutations. *Protein Sci*. 2021;30(1):60-69.

16. 1000 Genomes Project Consortium, Auton A, Brooks LD, et al. A global reference for human genetic variation. *Nature*. 2015;526(7571):68-74.

17. Siva N. 1000 genomes project. *Nat Biotechnol*. 2008;26(3):256.

18. Bray SM, Mulle JG, Dodd AF, Pulver AE, Wooding S, Warren ST. Signatures of founder effects, admixture, and selection in the Ashkenazi Jewish population. *Proc Natl Acad Sci U S A*. 2010;107(37):16222-16227.

19. Alexander DH, Lange K. Enhancements to the ADMIXTURE algorithm for individual ancestry estimation. *BMC Bioinformatics*. 2011;12:246.

20. Behr AA, Liu KZ, Liu-Fang G, Nakka P, Ramachandran S. pong: fast analysis and visualization of latent clusters in population genetic data. *Bioinformatics*. 2016;32(18):2817-2823.

21. Das S, Forer L, Schönherr S, et al. Next-generation genotype imputation service and methods. *Nat Genet*. 2016;48(10):1284-1287.

22. Hilmarsson H, Kumar AS, Rastogi R, Bustamante CD, Montserrat DM, Ioannidis AG. High resolution ancestry deconvolution for next generation genomic data. *bioRxiv*. Published online September 21, 2021. doi:10.1101/2021.09.19.460980

23. Martin M, Patterson M, Garg S, et al. WhatsHap: fast and accurate read-based phasing. *bioRxiv*. Published online November 2, 2016. doi:10.1101/085050

24. Conley AB, Rishishwar L, Ahmad M, et al. Rye: genetic ancestry inference at biobank scale. *Nucleic Acids Res*. 2023;51(8):e44.

25. Manichaikul A, Mychaleckyj JC, Rich SS, Daly K, Sale M, Chen WM. Robust relationship inference in genome-wide association studies. *Bioinformatics*. 2010;26(22):2867-2873.

26. Purcell S, Neale B, Todd-Brown K, et al. PLINK: a tool set for whole-genome association and population-based linkage analyses. *Am J Hum Genet*. 2007;81(3):559-575.

27. Simón-Sánchez J, Kilarski LL, Nalls MA, et al. Cooperative genome-wide analysis shows increased homozygosity in early onset Parkinson’s disease. *PLoS One*. 2012;7(3):e28787.

28. Nalls MA, Blauwendraat C, Vallerga CL, et al. Identification of novel risk loci, causal insights, and heritable risk for Parkinson’s disease: a meta-analysis of genome-wide association studies. *Lancet Neurol*. 2019;18(12):1091-1102.

29. Kim JJ, Vitale D, Otani DV, et al. Multi-ancestry genome-wide association meta-analysis of Parkinson’s disease. *Nat Genet*. 2024;56(1):27-36.

30. Loesch DP, Horimoto ARVR, Heilbron K, et al. Characterizing the Genetic Architecture of Parkinson’s Disease in Latinos. *Ann Neurol*. 2021;90(3):353-365.

31. Foo JN, Chew EGY, Chung SJ, et al. Identification of Risk Loci for Parkinson Disease in Asians and Comparison of Risk Between Asians and Europeans: A Genome-Wide Association Study. *JAMA Neurol*. 2020;77(6):746-754.

32. Bandrés-Ciga S, Price TR, Barrero FJ, et al. Genome-wide assessment of Parkinson’s disease in a Southern Spanish population. *Neurobiol Aging*. 2016;45:213.e3-e213.e9.

33. Dolzhenko E, Deshpande V, Schlesinger F, et al. ExpansionHunter: a sequence-graph-based tool to analyze variation in short tandem repeat regions. *Bioinformatics*. 2019;35(22):4754-4756.
