# Supplementary material for: Large-scale genetic characterization of Parkinson’s disease in the African and African admixed populations
Source: Brain. 2025 Oct 8;149(5):1537–53. doi: 10.1093/brain/awaf379 (PMC13140531; doi:10.1093/brain/awaf379)

Supplementary Figure 1. Ancestry prediction clustering for each cohort under study

Principal component analysis grouping individuals based on their genetic makeup. A) UK Biobank (UKB) B)All of Us C) GP2-BLAAC PD D) NPDRN E) PDGENE. Blue color indicates African ancestry, and green color indicates African admixed ancestry F) GP2-BLAAC PD, NPDRN, PDGENE samples projected onto the 1,000 Genomes Population data. Black color indicates known pathogenic or novel variant carriers. G) GP2-BLAAC PD, NPDRN, PDGENE samples projected onto the 1,000 Genomes Population African and African admixed subpopulations. Black color indicates known pathogenic or novel variant carriers. Reference panels include African Ancestry in Southwest USA (ASW), African Caribbean in Barbados (ACB), Gambian in the Western Division (GWD), Esan in Nigeria (ESN), Mende in Sierra Leone (MSL), Yoruba in Ibadan, Nigeria (YRI), and Luhya in Webuye, Kenya (LWK).

Supplementary Figure 2. Candidate variants identified in Parkinson's disease genes in individuals of African and African admixed ancestry.

*red highlighted: variants previously reported as pathogenic or likely pathogenic. For the recessive PD genes, variants only found in early-onset cases were included.

Supplementary Figure 3. *PRKN* B allele frequencies and log-R ratios from genotyping data.

Copy number variants (CNV) in *PRKN* were screened in 1,167 PD cases. A total of 872 African and 295 African admixed ancestry individuals were included in this study. Eight samples had positive CNV results for *PRKN* as shown in A) indicating a heterozygous deletion in exon 3, B) a heterozygous duplication in exon 2, C) a heterozygous deletion in exons 3 and 4 and a second potential heterozygous deletion in exon 4, D) a homozygous deletion in exons 3 and 4, E) a homozygous deletion in exons 3 and 4, F) a heterozygous duplication in exon 1, heterozygous deletion in exon 10, and a region of homozygosity in exons 2 and 3; G) a heterozygous duplication in exon 2, and H) a heterozygous deletion in exons 3 and 4. Reference Table 4 for demographic information.

Supplementary Figure 4. Predicted structural consequences of novel amino acid substitutions. Predicted structural consequences of novel amino acid substitutions visualized using AlphaFold and PyMOL v2.6.0. The analysis includes estimates of protein stability changes (ΔΔG), where more negative values suggest greater structural destabilization.

Supplementary Figure 5. Visualization of wild-type and expanded alleles using short-read sequencing data. Representative reads illustrate the difference in repeat structure between non-expanded and expanded alleles.

Supplementary Figure 6. Inferred ancestry proportions for individuals. Ancestry proportions from the GP2-BLAAC PD, NPDRN, and PDGENE datasets were inferred using ADMIXTURE at K=10.

Supplementary Figure 7. Local ancestry inference for prioritized variants identified in the GP2-BLAAC PD, NPDRN, and PDGENE datasets. a. Carriers of variants identified in the *GBA1* gene. b. Carriers of variants in *SNCA, PRKN, VPS35, FBXO7*, and *PLA2G6*.

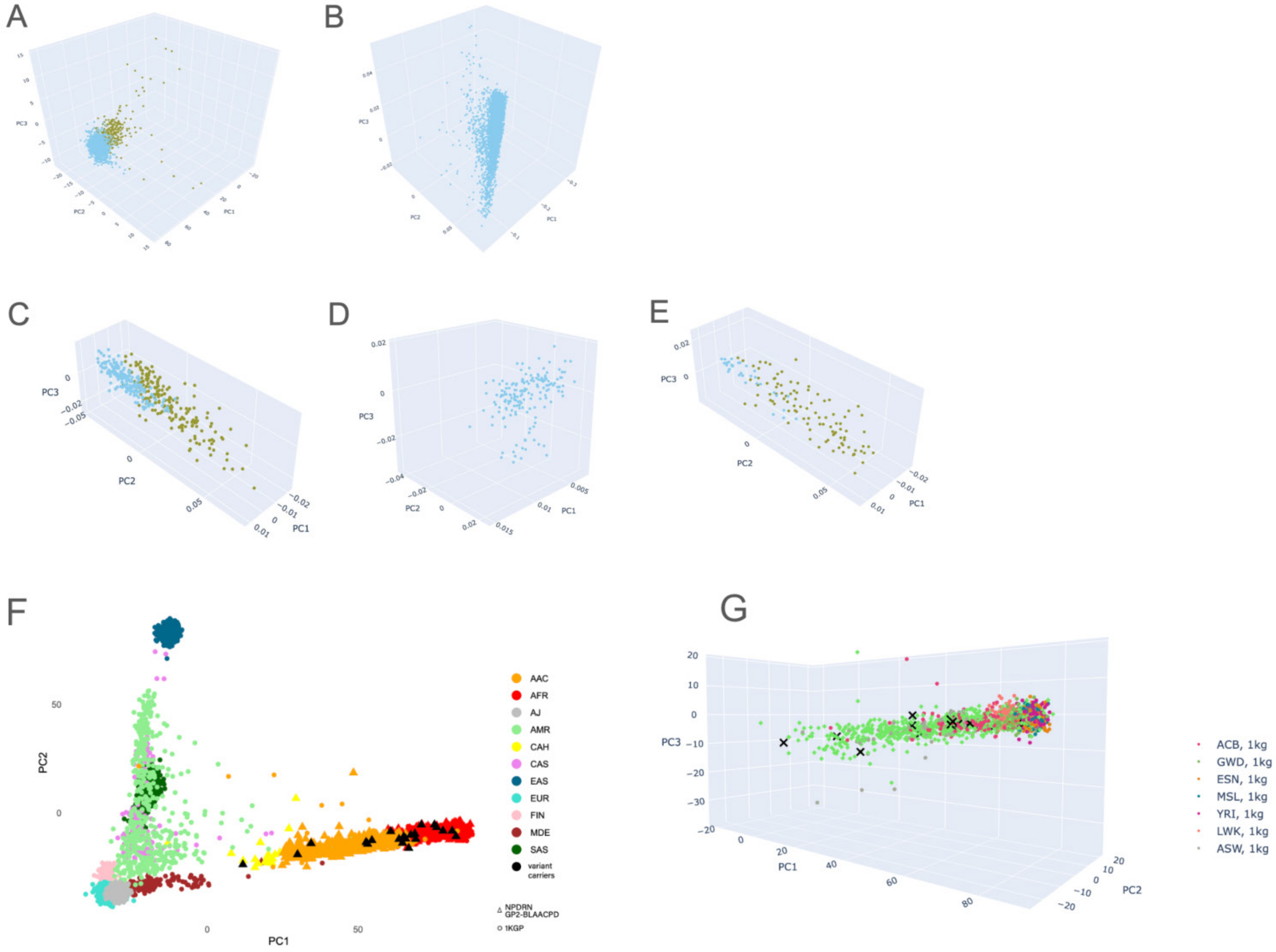

A
PC3
PC2
PC1
B
PC3
PC2
PC1
C
PC3
PC2
PC1
D
PC3
PC2
PC1
E
PC3
PC2
PC1
F
PC2
PC1
AAC
AFR
AJ
AMR
CAH
CAS
EAS
EUR
FIN
MDE
SAS
variant carriers
NPDRN GP2-BLAACPD
1KGP
G
PC3
PC1
PC2
ACB, 1kg
GWD, 1kg
ESN, 1kg
MSL, 1kg
YRI, 1kg
LWK, 1kg
ASW, 1kg

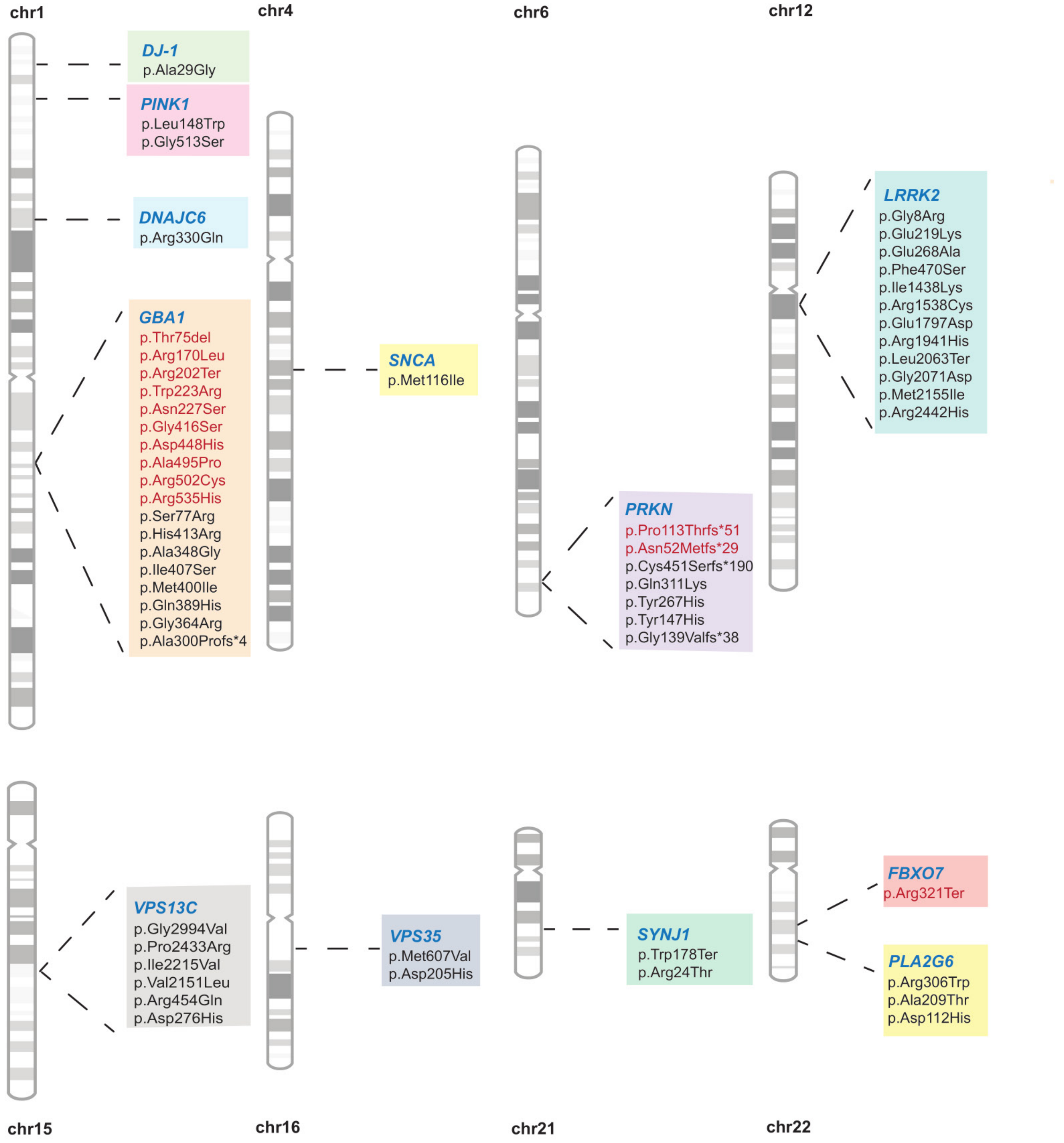
chr1
chr4
chr6
chr12
DJ-1
p.Ala29Gly
PINK1
p.Leu148Trp
p.Gly513Ser
DNAJC6
p.Arg330Gln
GBA1
p.Thr75del
p.Arg170Leu
p.Arg202Ter
p.Trp223Arg
p.Asn227Ser
p.Gly416Ser
p.Asp448His
p.Ala495Pro
p.Arg502Cys
p.Arg535His
p.Ser77Arg
p.His413Arg
p.Ala348Gly
p.Ile407Ser
p.Met400Ile
p.Gln389His
p.Gly364Arg
p.Ala300Profs*4
SNCA
p.Met116Ile
PRKN
p.Pro113Thrfs*51
p.Asn52Metfs*29
p.Cys451Serfs*190
p.Gln311Lys
p.Tyr267His
p.Tyr147His
p.Gly139Valfs*38
LRRK2
p.Gly8Arg
p.Glu219Lys
p.Glu268Ala
p.Phe470Ser
p.Ile1438Lys
p.Arg1538Cys
p.Glu1797Asp
p.Arg1941His
p.Leu2063Ter
p.Gly2071Asp
p.Met2155Ile
p.Arg2442His
VPS13C
p.Gly2994Val
p.Pro2433Arg
p.Ile2215Val
p.Val2151Leu
p.Arg454Gln
p.Asp276His
VPS35
p.Met607Val
p.Asp205His
SYNJ1
p.Trp178Ter
p.Arg24Thr
FBXO7
p.Arg321Ter
PLA2G6
p.Arg306Trp
p.Ala209Thr
p.Asp112His
chr15
chr16
chr21
chr22

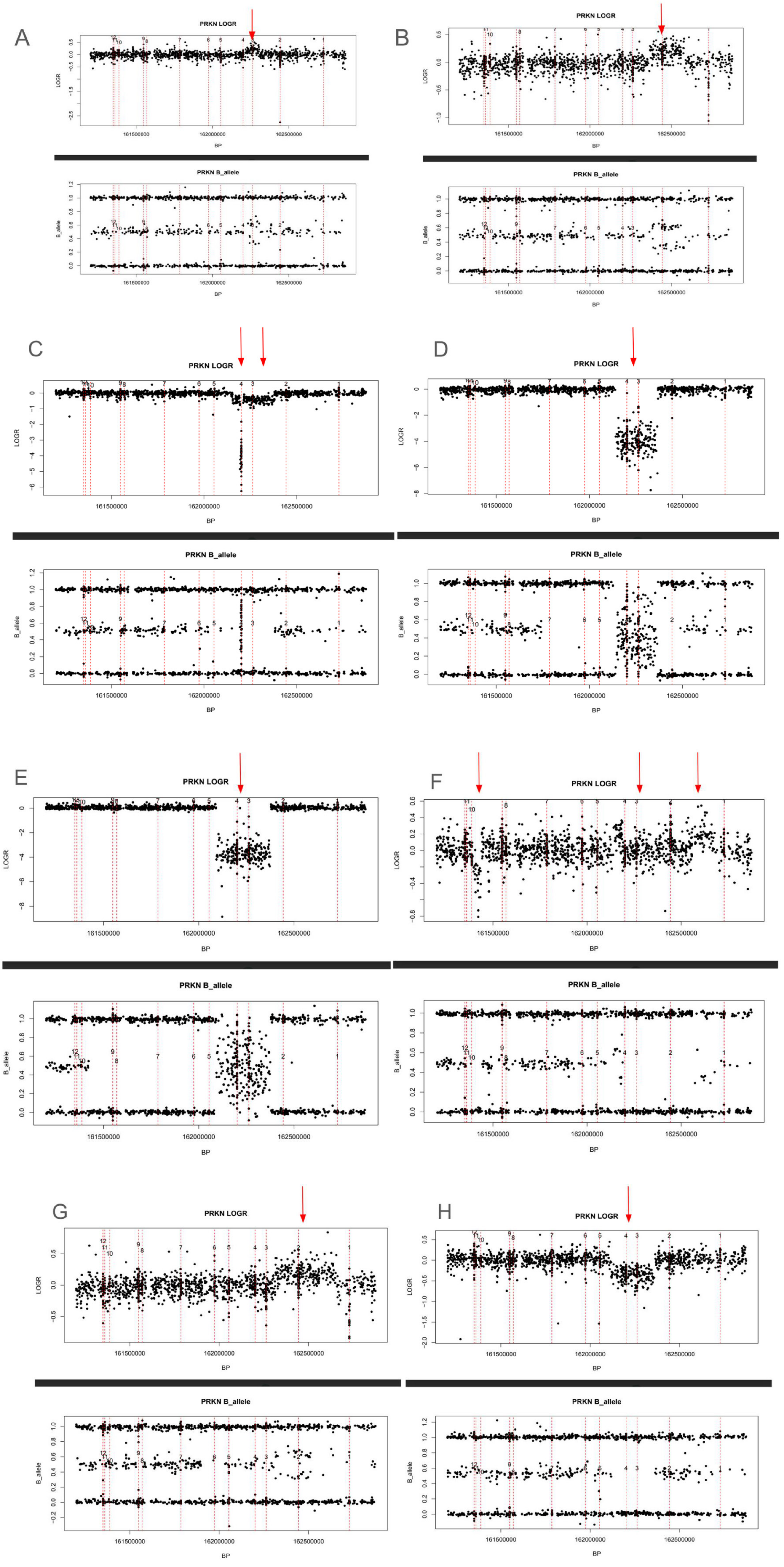
A
PRKN LOGR
LOGR
BP
PRKN B_allele
B_allele
BP
B
PRKN LOGR
LOGR
BP
PRKN B_allele
B_allele
BP
C
PRKN LOGR
LOGR
BP
PRKN B_allele
B_allele
BP
D
PRKN LOGR
LOGR
BP
PRKN B_allele
B_allele
BP
E
PRKN LOGR
LOGR
BP
PRKN B_allele
B_allele
BP
F
PRKN LOGR
LOGR
BP
PRKN B_allele
B_allele
BP
G
PRKN LOGR
LOGR
BP
PRKN B_allele
B_allele
BP
H
PRKN LOGR
LOGR
BP
PRKN B_allele
B_allele
BP
161500000
162000000
162500000

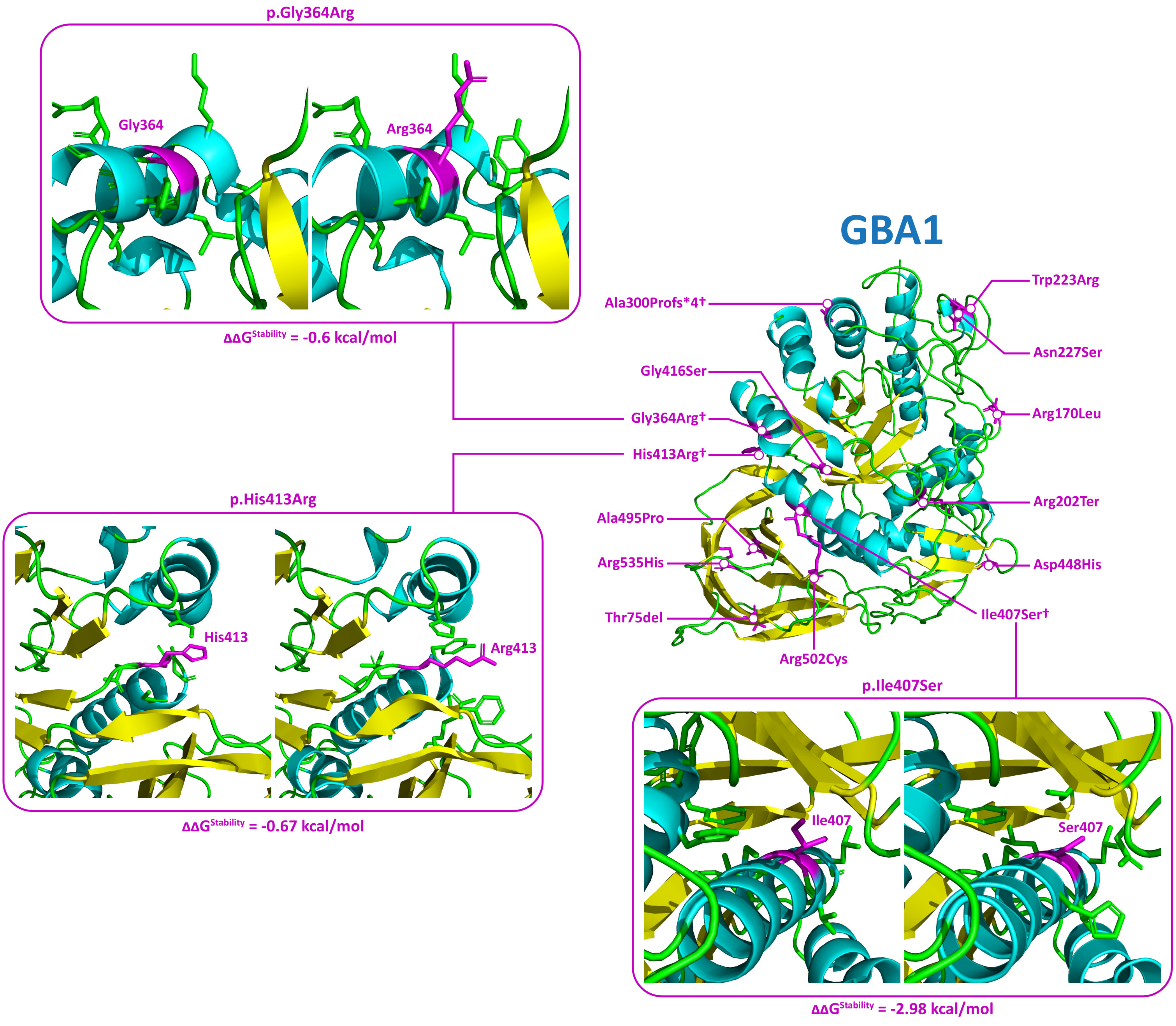

p.Gly364Arg
Gly364
Arg364
ΔΔG^Stability = -0.6 kcal/mol
GBA1
Ala300Profs*4†
Trp223Arg
Asn227Ser
Gly416Ser
Arg170Leu
Gly364Arg†
His413Arg†
Arg202Ter
Ala495Pro
Arg535His
Asp448His
Thr75del
Ile407Ser†
Arg502Cys
p.His413Arg
His413
Arg413
ΔΔG^Stability = -0.67 kcal/mol
p.Ile407Ser
Ile407
Ser407
ΔΔG^Stability = -2.98 kcal/mol

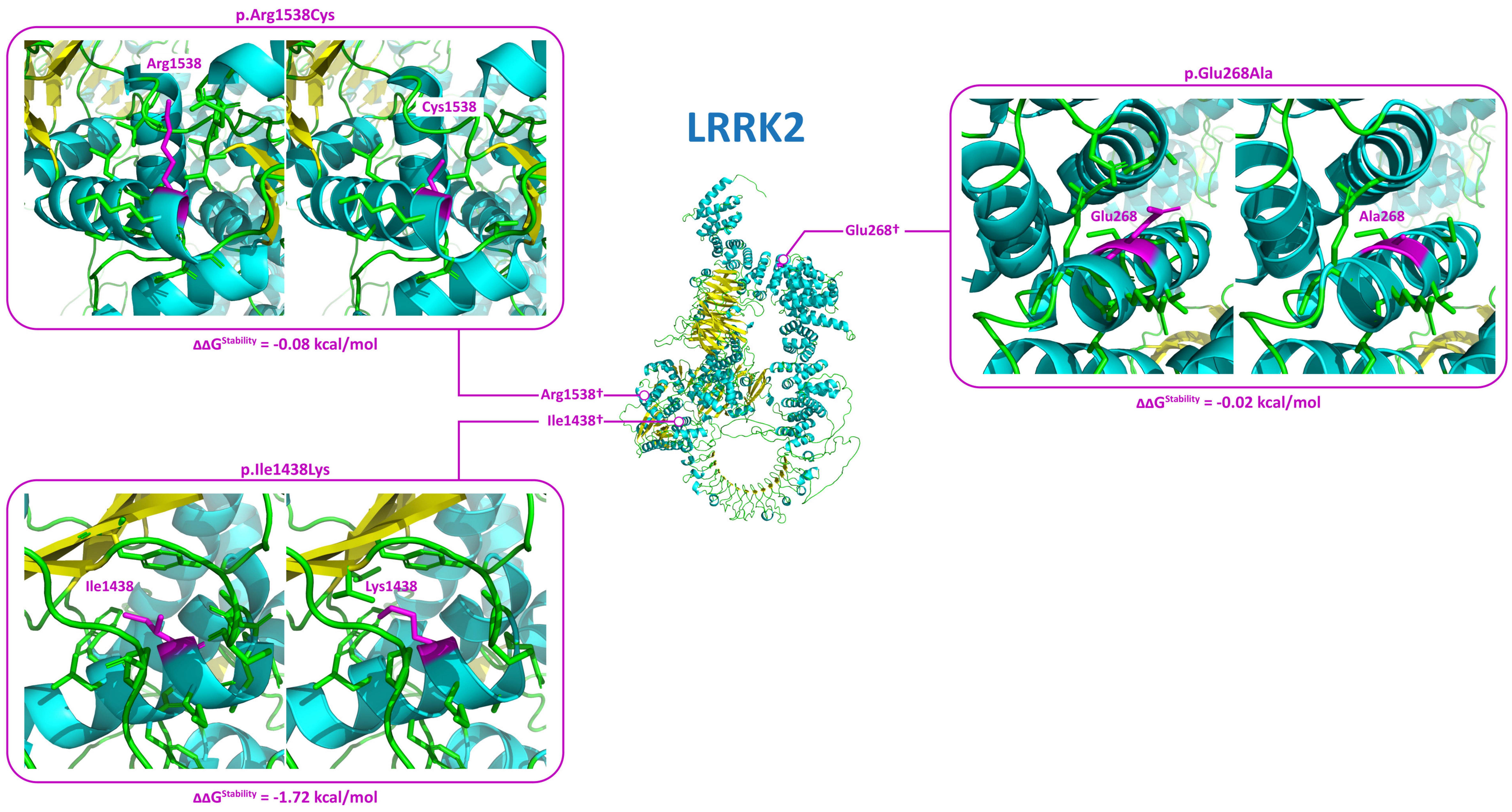

p.Arg1538Cys
Arg1538
Cys1538
ΔΔG Stability = -0.08 kcal/mol
LRRK2
Arg1538†
Ile1438†
Glu268†
p.Glu268Ala
Glu268
Ala268
ΔΔG Stability = -0.02 kcal/mol
p.Ile1438Lys
Ile1438
Lys1438
ΔΔG Stability = -1.72 kcal/mol

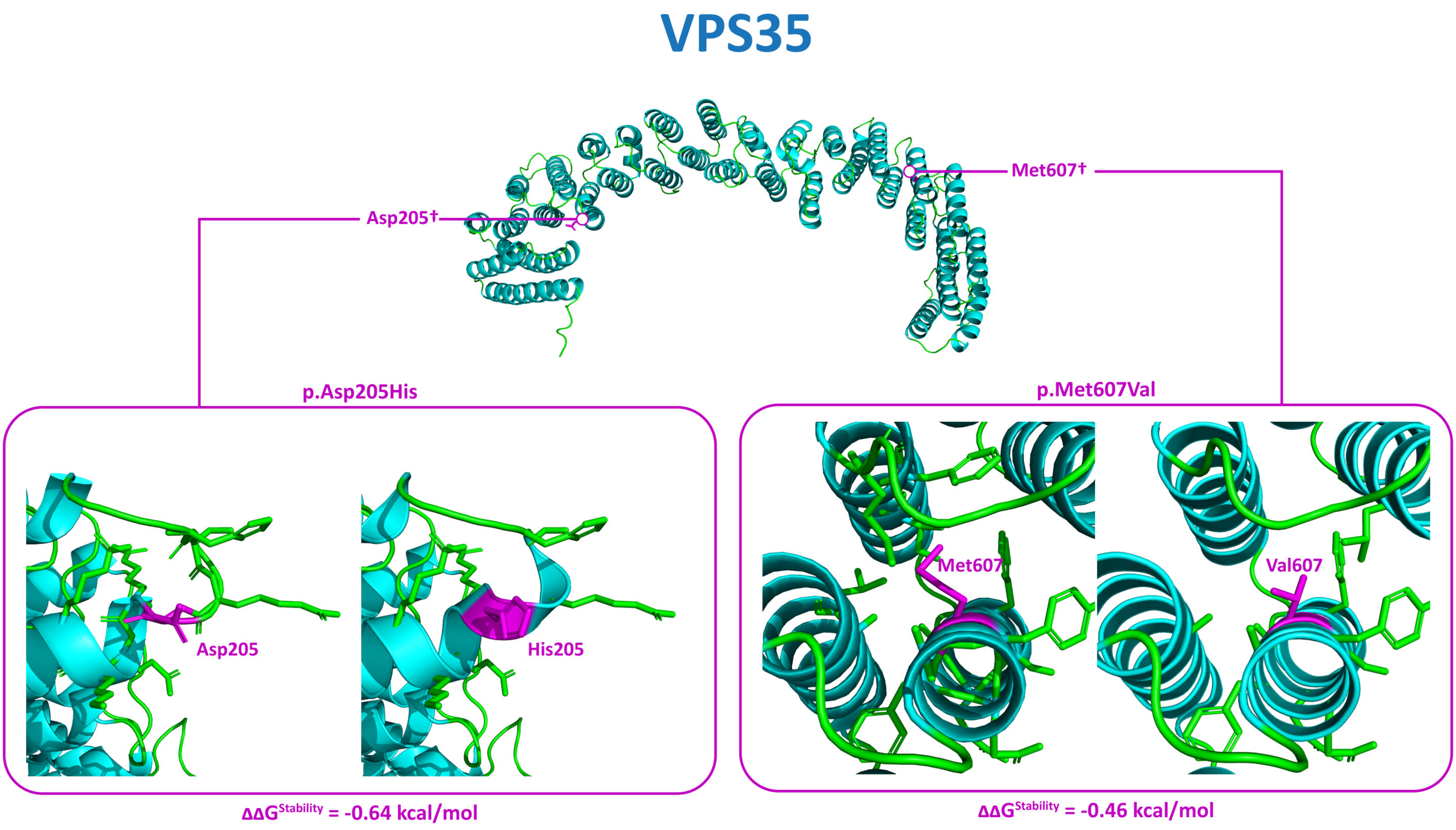
VPS35
Asp205†
Met607†
p.Asp205His
Asp205
His205
p.Met607Val
Met607
Val607
ΔΔG^Stability = -0.64 kcal/mol
ΔΔG^Stability = -0.46 kcal/mol

SNCA

p.Met116Ile

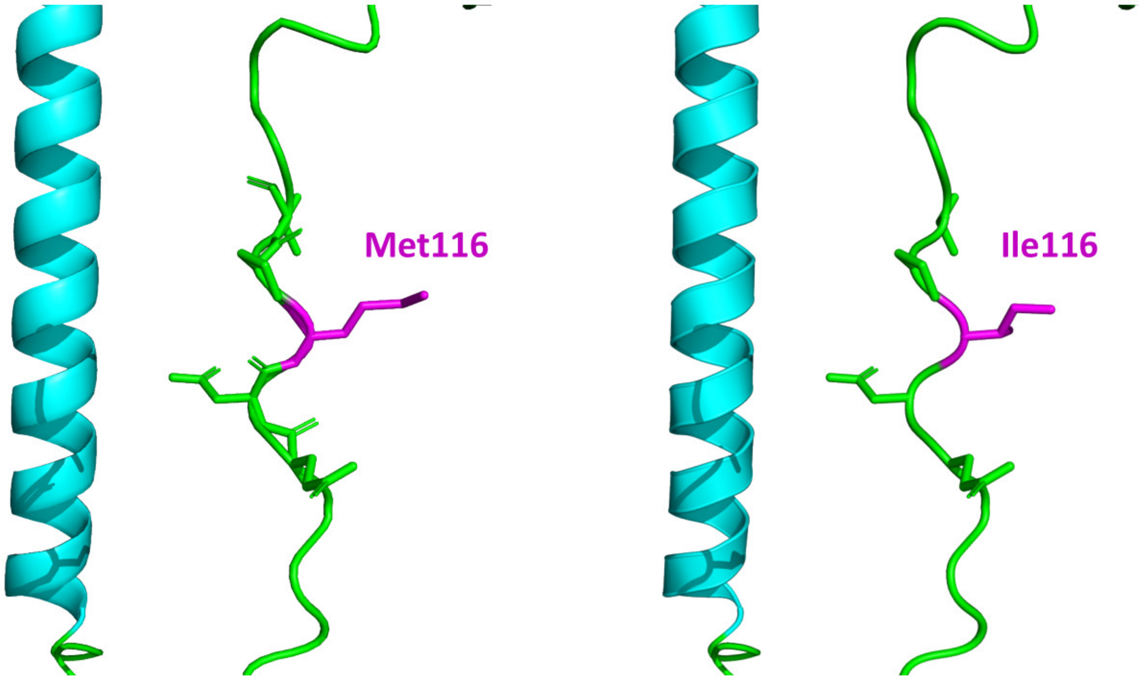

$\Delta\Delta G^{Stability}$ = 0.12 kcal/mol

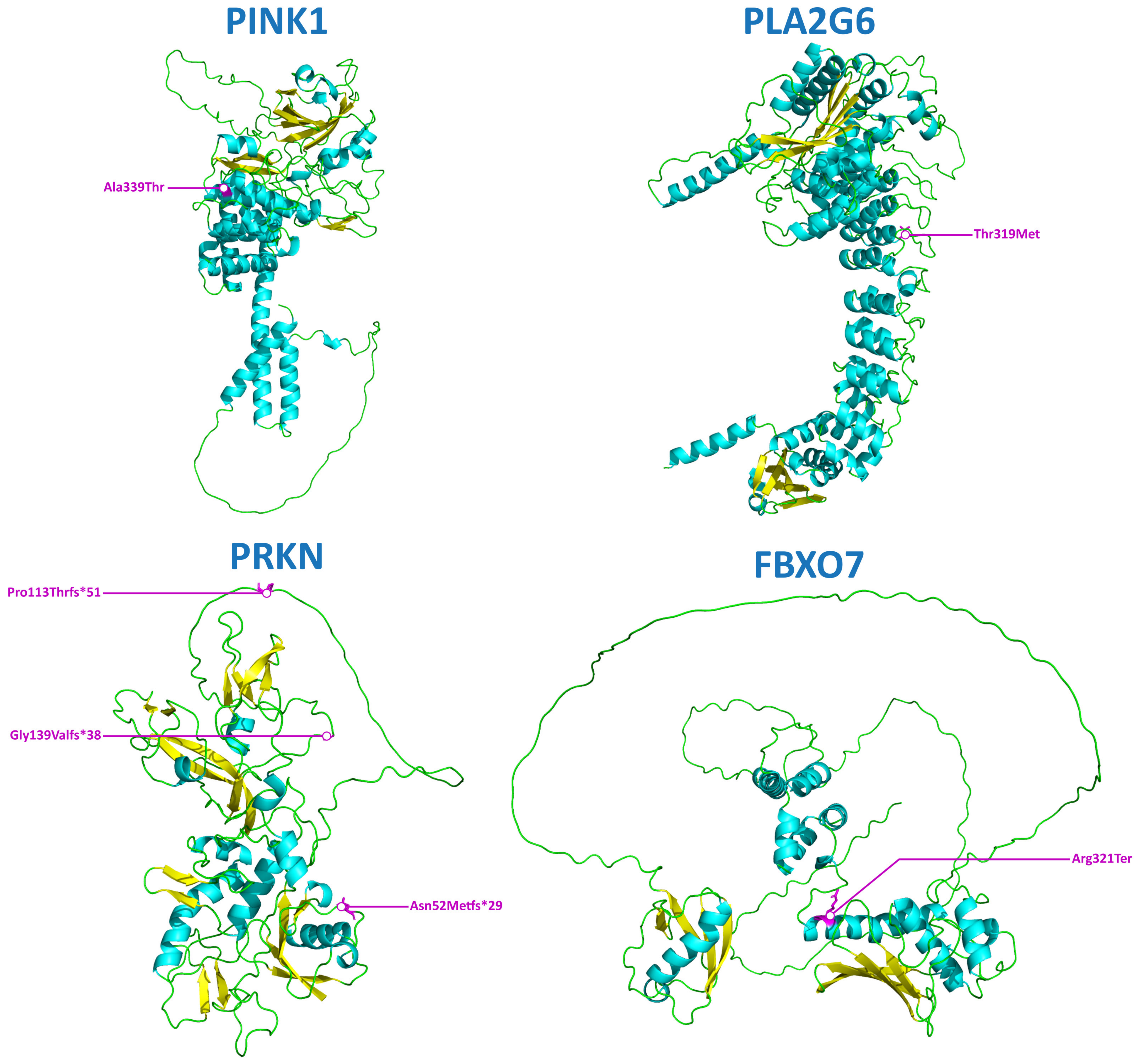
PINK1
Ala339Thr
PLA2G6
Thr319Met
PRKN
Pro113Thrfs*51
Gly139Valfs*38
Asn52Metfs*29
FBXO7
Arg321Ter

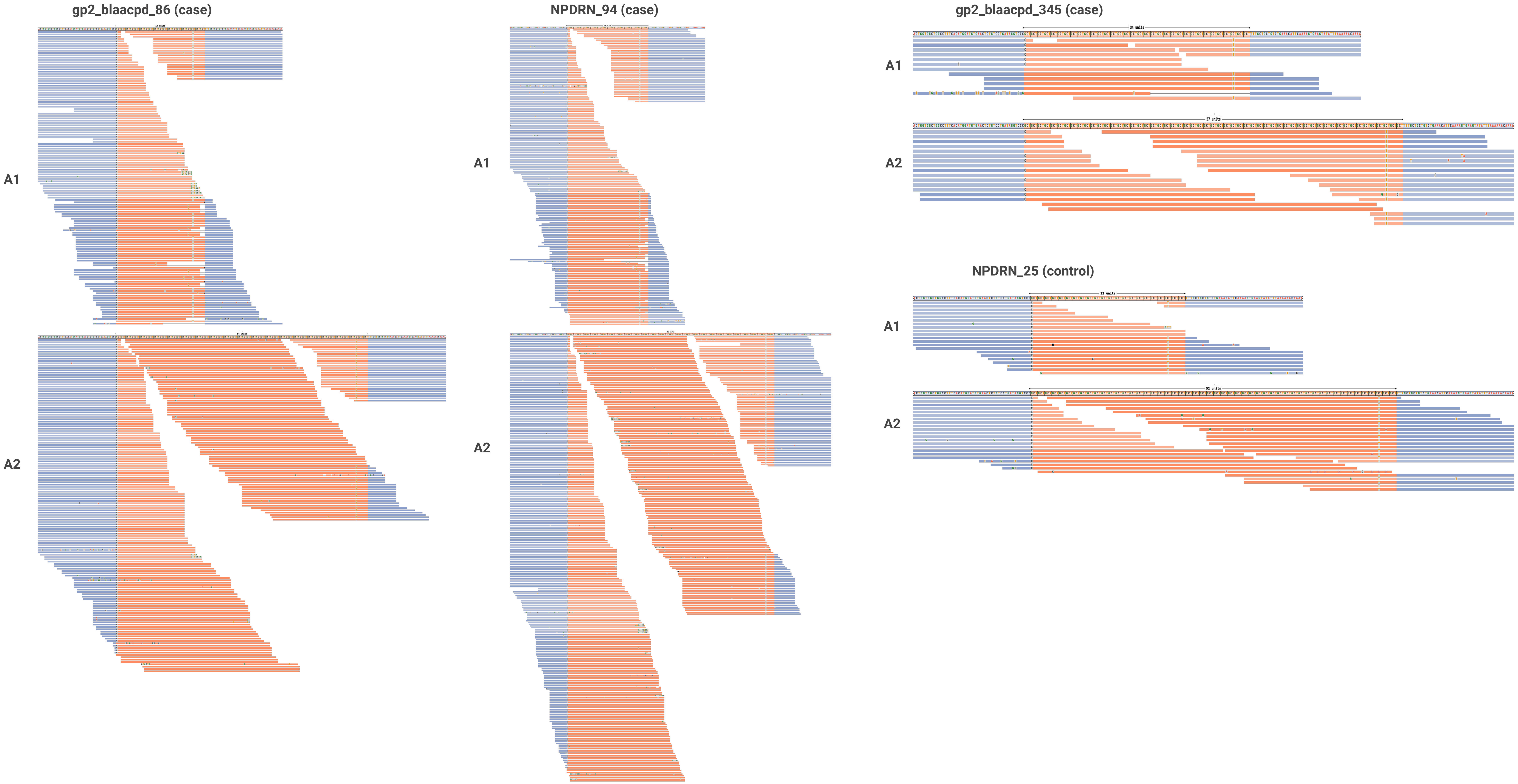
gp2_blaacpd_86 (case)
NPDRN_94 (case)
gp2_blaacpd_345 (case)
NPDRN_25 (control)
A1
A2
A1
A2
A1
A2
A1
A2

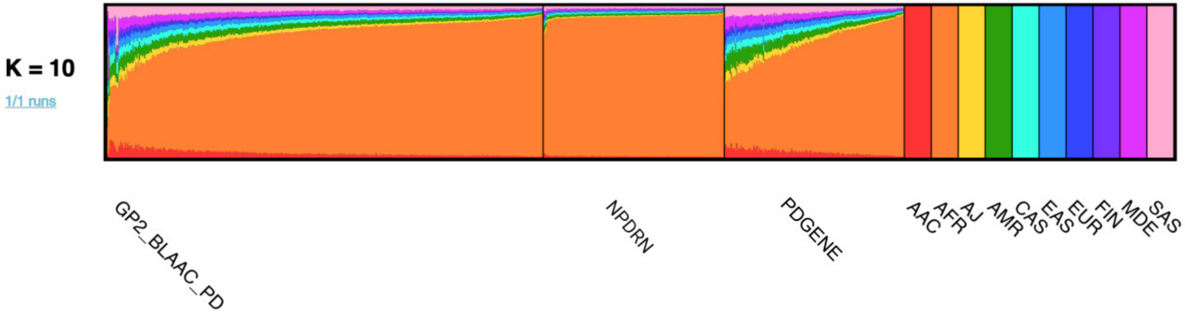
K = 10
1/1 runs
GP2_BLAAC_PD
NPDRN
PDGENE
AAC
AFR
AJ
AMR
CAS
EAS
EUR
FIN
MDE
SAS

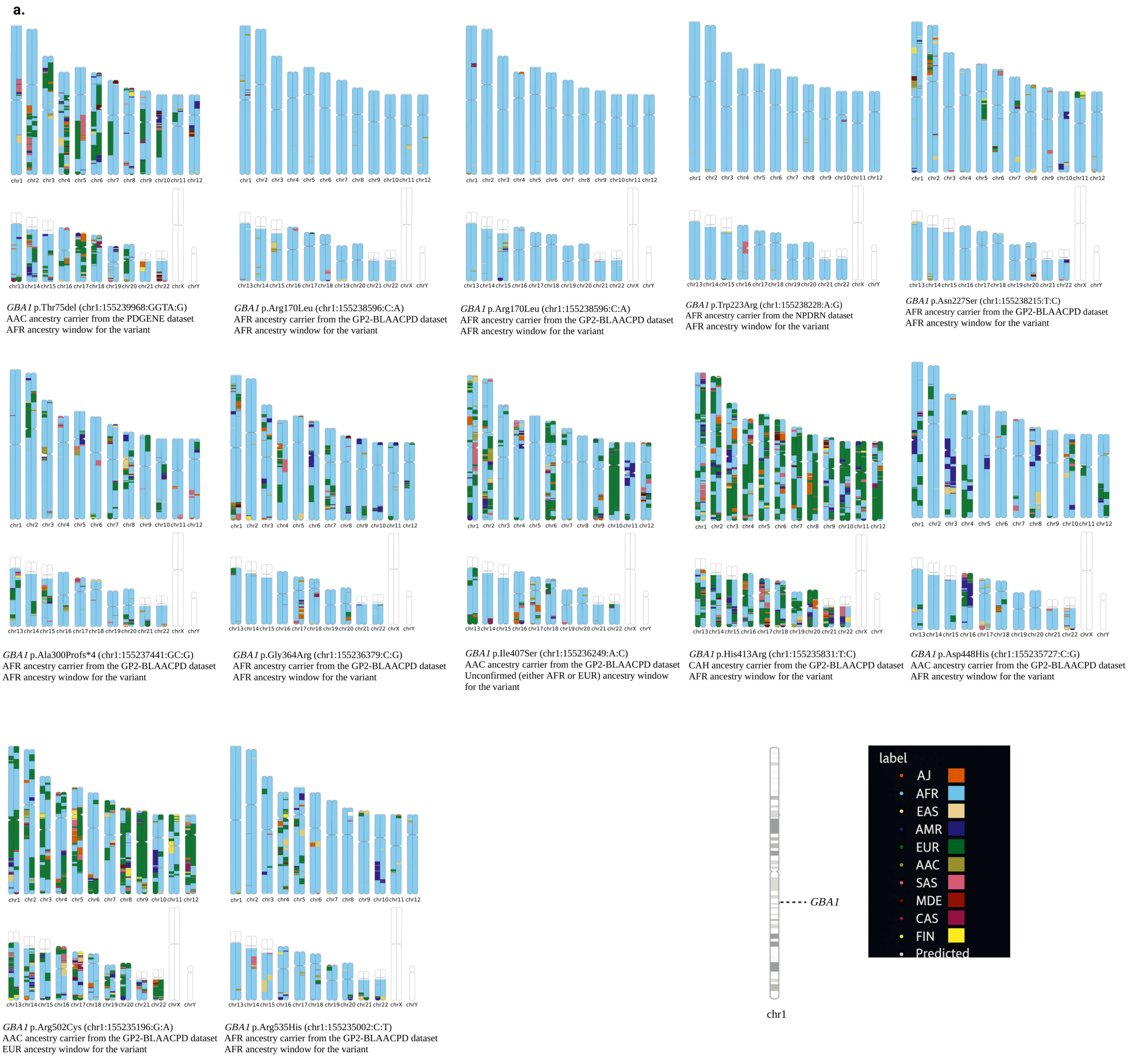
a.
GBA1 p.Thr75del (chr1:155239968:GGTA:G)
AAC ancestry carrier from the PDGENE dataset
AFR ancestry window for the variant
GBA1 p.Arg170Leu (chr1:155238596:C:A)
AFR ancestry carrier from the GP2-BLAACPD dataset
AFR ancestry window for the variant
GBA1 p.Arg170Leu (chr1:155238596:C:A)
AFR ancestry carrier from the GP2-BLAACPD dataset
AFR ancestry window for the variant
GBA1 p.Trp223Arg (chr1:155238228:A:G)
AFR ancestry carrier from the NPDRN dataset
AFR ancestry window for the variant
GBA1 p.Asn227Ser (chr1:155238215:T:C)
AFR ancestry carrier from the GP2-BLAACPD dataset
AFR ancestry window for the variant
GBA1 p.Ala300Profs*4 (chr1:155237441:GC:G)
AFR ancestry carrier from the GP2-BLAACPD dataset
AFR ancestry window for the variant
GBA1 p.Gly364Arg (chr1:155236379:C:G)
AFR ancestry carrier from the GP2-BLAACPD dataset
AFR ancestry window for the variant
GBA1 p.Ile407Ser (chr1:155236249:A:C)
AAC ancestry carrier from the GP2-BLAACPD dataset
Unconfirmed (either AFR or EUR) ancestry window for the variant
GBA1 p.His413Arg (chr1:155235831:T:C)
CAH ancestry carrier from the GP2-BLAACPD dataset
AFR ancestry window for the variant
GBA1 p.Asp448His (chr1:155235727:C:G)
AAC ancestry carrier from the GP2-BLAACPD dataset
AFR ancestry window for the variant
GBA1 p.Arg502Cys (chr1:155235196:G:A)
AAC ancestry carrier from the GP2-BLAACPD dataset
EUR ancestry window for the variant
GBA1 p.Arg535His (chr1:155235002:C:T)
AFR ancestry carrier from the GP2-BLAACPD dataset
AFR ancestry window for the variant
GBA1
chr1
label
AJ
AFR
EAS
AMR
EUR
AAC
SAS
MDE
CAS
FIN
Predicted

b.

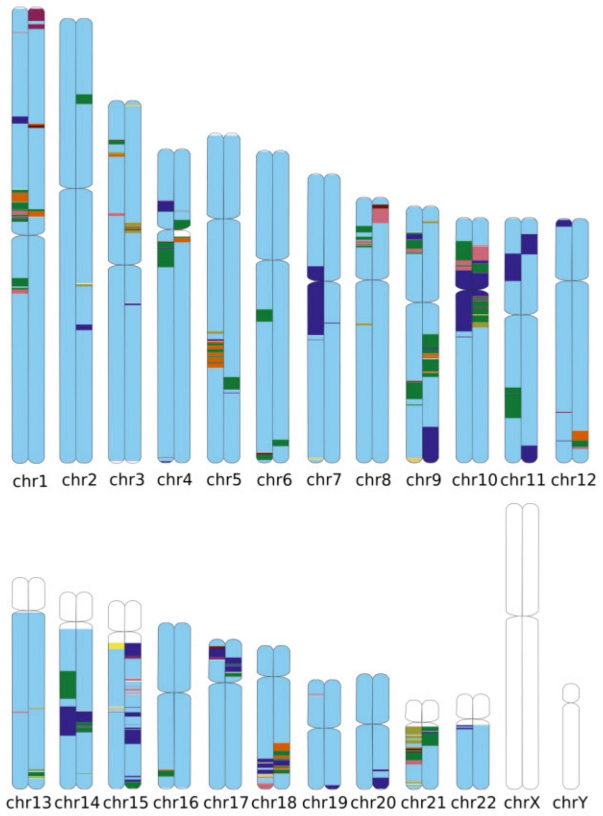

SNCA p.Met116Ile (chr4:89729236:C:A)
AFR ancestry carrier from the PDGENE dataset
AFR ancestry window for the variant

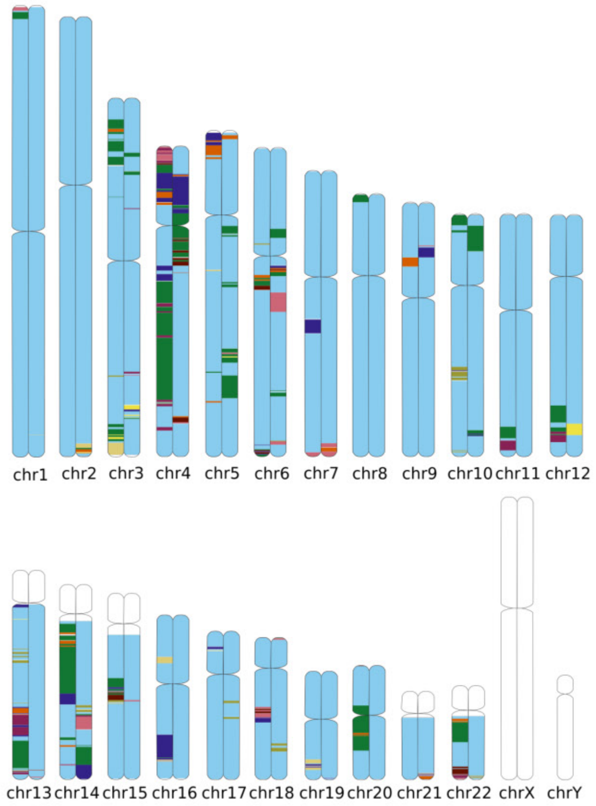

PRKN p.Asn52Metfs*29 (chr6:162443325:AT:A)
AAC ancestry carrier from the PDGENE dataset
SAS ancestry window for the variant

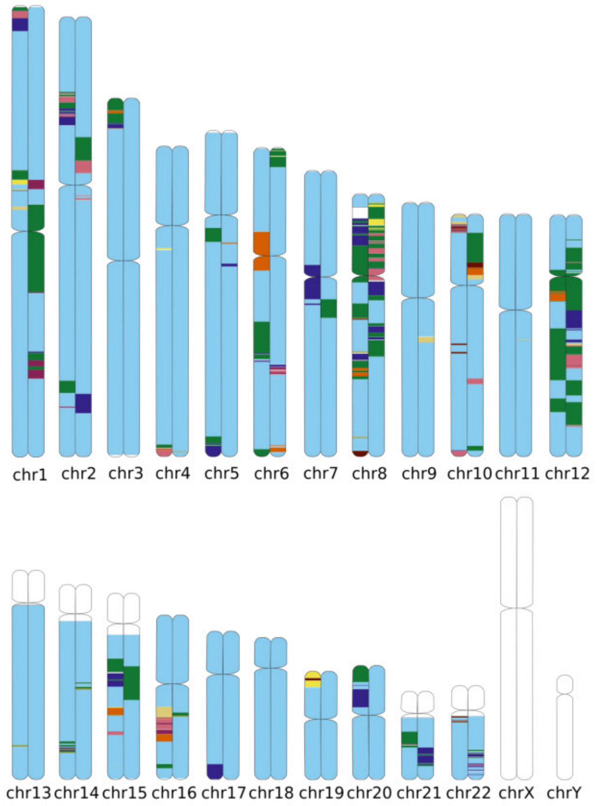

PRKN p.Pro113Thrfs*51 (chr6:6:162262560)
AFR ancestry carrier from the PDGENE dataset
Unconfirmed ancestry window for the deletion variant

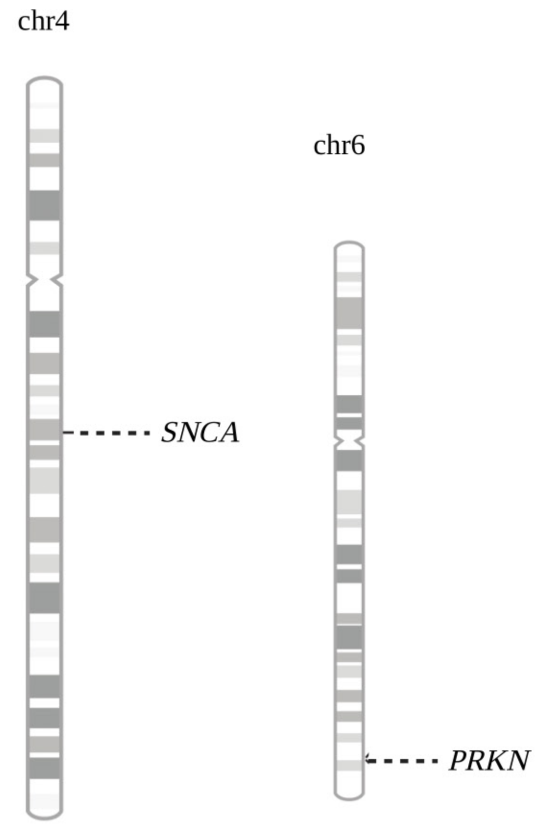

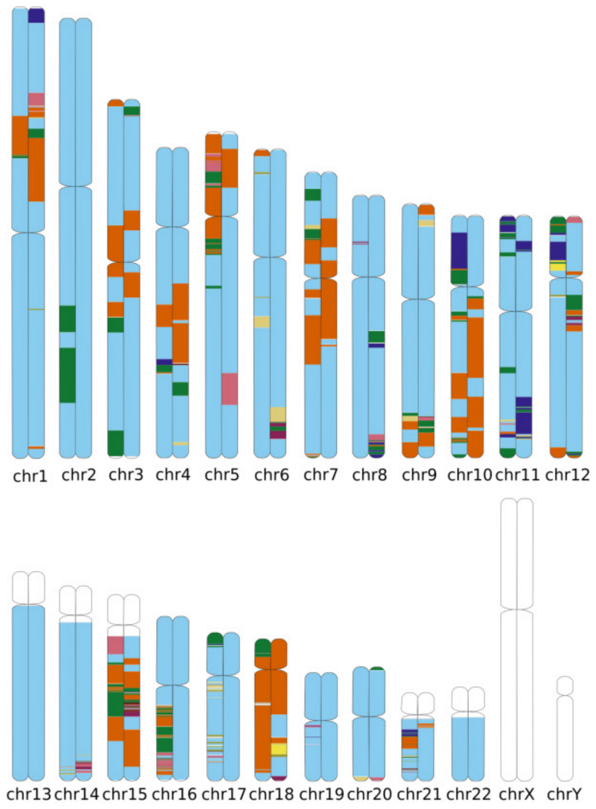

VPS35 p.Asp205His (chr16:46679050:C:G)
AAC ancestry carrier from the GP2-BLAACPD dataset
AFR ancestry window for the variant

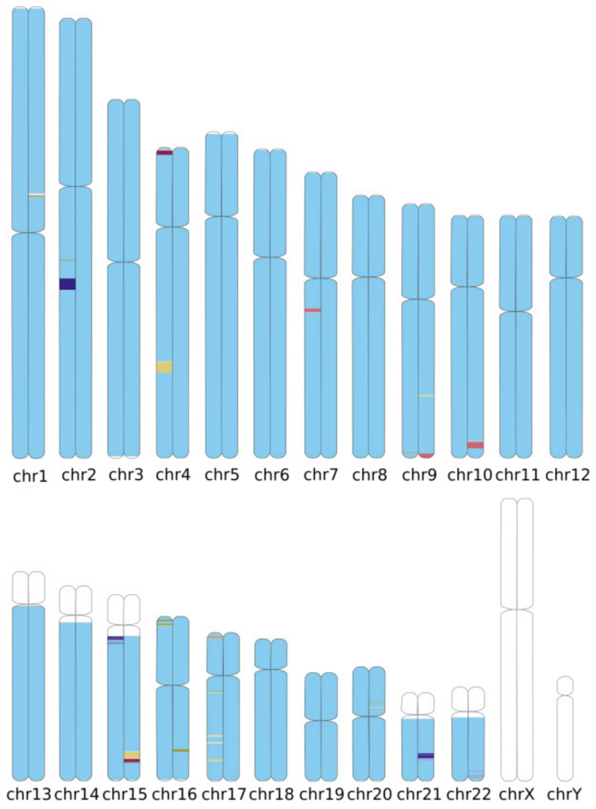

VPS35 p.Met607Val (chr16:46662991:T:C)
AFR ancestry carrier from the NPDRN dataset
AFR ancestry window for the variant

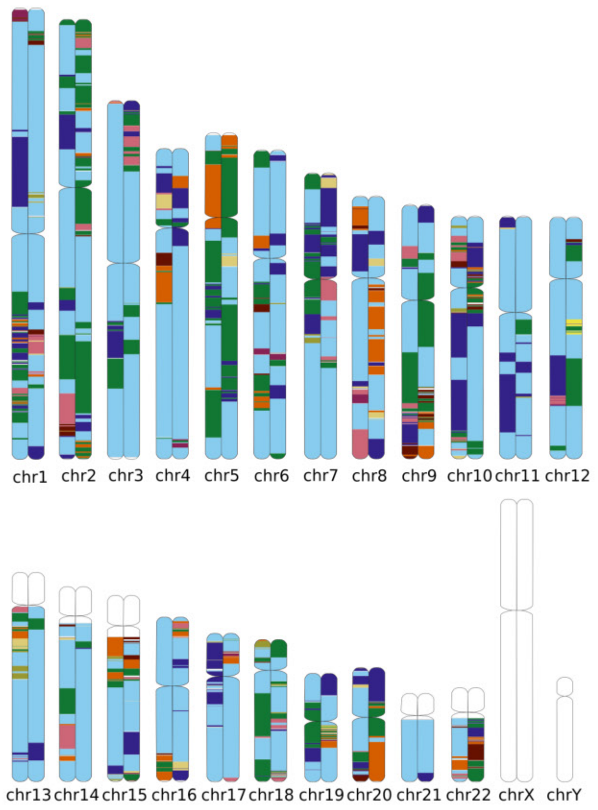

FBXO7 p.Arg321Ter (chr22:32491175:C:T)
AAC ancestry carrier from the PDGENE dataset
MDE ancestry window for the variant

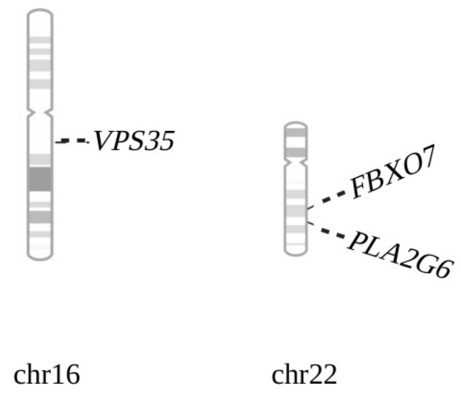

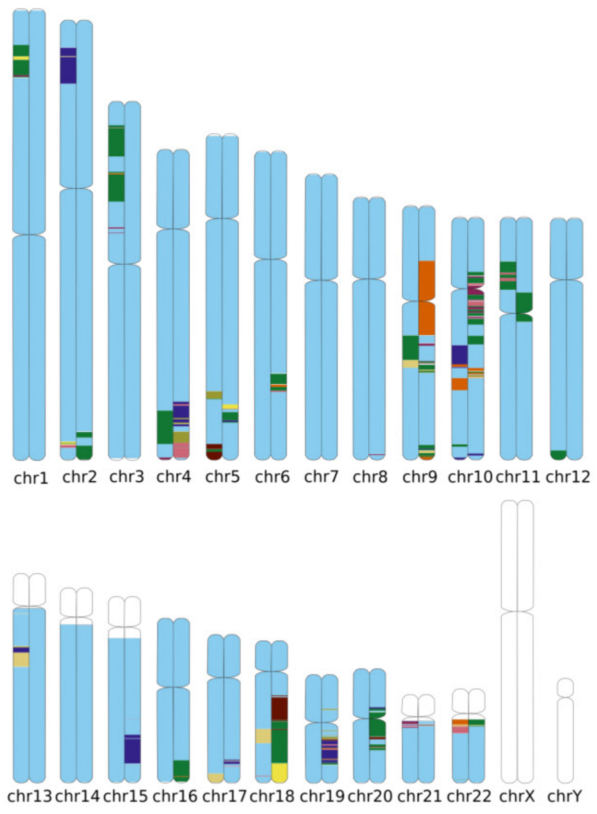

PLA2G6 p.Thr319Met (chr22:38132952:G:A)
AFR ancestry carrier from the PDGENE dataset
AFR ancestry window for the variant

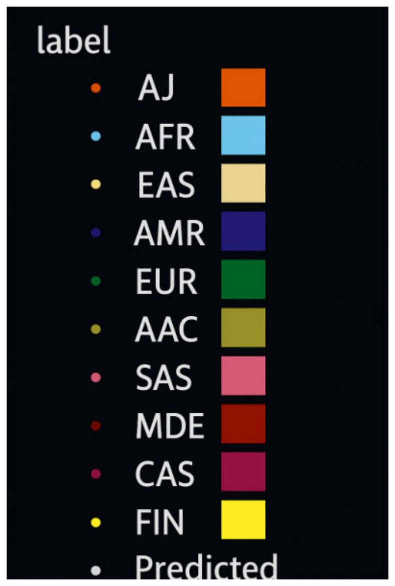

Supplement: awaf379_Supplementary_Data [file awaf379_supplementary_data.zip › brain-2025-00177-File010.pdf]
